# Genome-wide identification and expression profiling of the *GYF* gene family in *Vanilla planifolia*: insights into fruit development and Cymbidium mosaic virus response

**DOI:** 10.3389/fpls.2026.1795375

**Published:** 2026-04-17

**Authors:** Jesse Potts, Tao Jiang, Vincent N. Michael, Heqiang Huo, Xingbo Wu

**Affiliations:** 1 Department of Horticultural Sciences, Tropical Research and Education Center, IFAS, University of Florida, Homestead, FL, United States; 2 Mid-Florida Research and Education Center, IFAS, University of Florida, Apopka, FL, United States; 3 Crop Transformation Center, IFAS, University of Florida, Gainesville, FL, United States; 4 Department of Horticultural Sciences, IFAS, University of Florida, Gainesville, FL, United States

**Keywords:** *Cymbidium* mosaic virus, EXA1, GYF domain, potexvirus, susceptibility factor, *Vanilla planifolia*

## Abstract

**Introduction:**

*Vanilla planifolia* is a tropical crop producing high-value natural vanilla flavor but suffers chronic yield losses from Cymbidium mosaic virus (CymMV), a potexvirus for which resistance sources are limited and durable genetic resistance is not currently available. GYF (glycine-tyrosine-phenylalanine) domain proteins regulate mRNA surveillance, translational control, and immunity across eukaryotes. In Arabidopsis, the GYF protein EXA1 functions as a potexvirus susceptibility factor; however, no genome-wide analysis of the GYF gene family has been reported for *V. planifolia*.

**Methods:**

We performed genome-wide identification of GYF genes in the haplotype-phased *V. planifolia* genome using HMM profiling, BLASTP, and domain annotation. Phylogenetic, syntenic, and promoter cis-element analyses were conducted. Transcriptome data from fruits of high- and low-vanillin accessions were analyzed. CymMV inoculation experiments were performed over nine weeks, with viral accumulation confirmed by coat-protein transcript quantification and gene expression monitored by qRT-PCR.

**Results:**

We identified 11 GYF loci represented by 22 haplotype-specific allelic copies (VpGYF1–VpGYF22). Phylogenetic analysis grouped them into three clades corresponding to ATXR3/ATXR7 SET–GYF chromatin regulators (Clade I), NERD-like multi-domain proteins (Clade II), and EXA1-like translational regulators (Clade III). Ka/Ks ratios indicated purifying selection. Promoter analysis identified abundant SA-, MeJA-, and ethylene-responsive elements alongside WRKY, MYB, and MYC/bHLH sites. VpGYF expression in fruits correlated with vanillin content, with higher expression in glucovanillin-rich inner white tissue. Following CymMV inoculation, VpGYF genes showed broad transcriptional responsiveness, with the strongest induction in VpGYF4/GYF12, VpGYF19/GYF21, and VpGYF6/GYF14.

**Discussion:**

These results implicate VpGYF genes in both secondary metabolism and potexvirus host interactions in vanilla. The infection-responsive loci are prioritized as candidates for functional validation and host factor–based resistance strategies.

## Introduction

The GYF domain is a protein–protein interaction module conserved across eukaryotes. The domain takes its name from the Gly-Tyr-Phe tripeptide within its ligand-binding surface, which adopts a bulge-helix-bulge fold that recognizes proline-rich sequences matching the PPGϕ consensus (Freund, 2004; M. M. Kofler and Freund, 2006). Two major subfamilies exist: CD2BP2-type proteins localize to the nucleus and participate in pre-mRNA splicing, whereas SMY2-type proteins function in the cytoplasm to regulate mRNA surveillance, translational control, and vesicular transport (Ash et al., 2010; M. Kofler et al., 2009). SMY2-type GYF proteins in other eukaryotes (e.g., mammals) associate with cap-binding factors and deadenylase complexes to repress translation and aid mRNA decay (Morita et al., 2012; Christie and Igreja, 2023). Plant GYF proteins remain poorly characterized. Although previous studies have examined GYF-domain proteins in *Arabidopsis* and *Brassica species* (Wu et al., 2017; Zhang et al., 2023), no genome-wide analysis has been reported for vanilla or orchid species.

*Arabidopsis thaliana* encodes eight GYF proteins distributed across distinct functional categories (Wu et al., 2017). Among them, *EXA1* (Essential for potexvirus Accumulation 1) emerged as a host factor required for potexvirus infection (Hashimoto et al., 2016a; Yusa et al., 2019). *EXA1* interacts with eIF4E-family translation factors (eukaryotic initiation factor 4E) to facilitate potexvirus infection and acts as a susceptibility factor (Nishikawa et al., 2023). It also negatively regulates NLR immune receptor accumulation through translational repression as *exa1* mutants display enhanced disease resistance and elevated NLR protein levels (Wu et al., 2017). Virus-induced gene silencing of *NbEXA1* in *Nicotiana benthamiana* compromised the accumulation of *Plantago asiatica* mosaic virus (PlAMV) and Potato virus X (PVX) and silencing the tomato ortholog impeded Pepino mosaic virus infection (Hashimoto et al., 2016a). Rice encodes *EXA1*-like GYF proteins, but their roles in monocot–virus interactions remain uncharacterized (Yusa et al., 2019). These observations suggest that *EXA1*-mediated susceptibility mechanisms are conserved across eudicots. *EXA1* thus bridges viral components to the host translational machinery, functioning as a susceptibility factor that can be targeted to confer viral resistance (Hashimoto et al., 2016a, 2016b).

The remaining *Arabidopsis* GYF proteins occupy different regulatory niches. *PSIG1* (Plant SMY2-type Ile-GYF domain-containing protein 1) dampens programmed cell death during pathogen challenge by interacting with the NMD factor *SMG7* in cytoplasmic P-bodies (Matsui et al., 2017). *NERD* (Needed for *RDR2*-independent DNA methylation) couples small-RNA pathways to DNA methylation and RNA silencing through its GYF, SWIB, PHD, and Plus-3 domains (Pontier et al., 2012). *ATXR3* (also known as *SDG2*) and *ATXR7* (*SDG25*) are SET domain-containing H3K4 methyltransferases that fuse GYF domains with chromatin-modifying activities; both regulate defense-gene expression and plant immunity via epigenetic mechanisms (Berr et al., 2010; Lee et al., 2016). The *GYF* gene family thus spans viral susceptibility, chromatin modification, RNA metabolism, and immune regulation. Despite this functional breadth, systematic genome-wide identification of GYF genes has been limited to Brassica species (Zhang et al., 2023). Understanding the whole repertoire, evolutionary relationships, and domain architectures of GYF proteins in other plant lineages, particularly monocots, requires comprehensive genome-wide analyses which have not yet been conducted.

Vanilla (*Vanilla planifolia*) is the source of natural vanilla extract, the world’s second-most valuable spice, but heavily impacted by Cymbidium mosaic virus (CymMV). CymMV belongs to the Potexvirus genus and infects orchids worldwide. The virus produces chlorotic and necrotic patterns on leaves and flowers and is systemic. Infected plants harbor virus throughout the roots, bulbs, leaves, and flowers (Hanssen and Thomma, 2010; Zettler et al., 1990; Yusop et al., 2022). In vanilla, the chronic CymMV infection causes chlorotic mottling and necrotic flecking, resulting in reduced plant vigor and severe production decline (Gu et al., 2024). It was found to be massively presented within the fruits and leaves of vanilla plants and recognized as one of the most devastating viruses causing losses in the vanilla industry. The vegetative propagation of vanilla cuttings in commercial production increases the risk of virus transmission through infected mother plants. Despite the relatively high genetic diversity reported within Vanilla species, durable resistance sources have not yet been identified for commercial *V. planifolia*. Genetic engineering of susceptibility factors, such as GYF-domain proteins, represents a promising approach for improving virus resistance in vanilla.

The recent release of a haplotype-phased, chromosome-scale genome assembly for *V. planifolia* (Hasing et al., 2020; Piet et al., 2022), enables genome-wide analyses that were previously impossible in this crop. We exploited this resource to conduct a systematic identification of the *GYF* gene family in vanilla by identifying all GYF domain-containing genes, analyzing their phylogenetic relationships with GYF proteins from Arabidopsis, rice, tomato, and other orchids, and characterizing their conserved domains, gene structures, chromosomal distribution, syntenic relationships, and promoter *cis*-elements. We examined *VpGYF* expressions during fruit development using transcriptome analyses of high- and low-vanillin accessions and monitored transcriptional responses to controlled CymMV inoculation over nine weeks by qRT-PCR. Our results show that *VpGYF* genes partition into three phylogenetic clades matching the functional categories defined in *Arabidopsis*, exhibit tissue-specific expression patterns associated with fruit development, and display broad transcriptional responsiveness following potexvirus challenge, with the strongest infection-associated induction observed for *VpGYF4/GYF12*, *VpGYF19/GYF21*, and *VpGYF6/GYF14*. The temporal association between *VpGYF* expression patterns and viral accumulation suggests that some GYF-domain proteins may participate in host processes associated with infection; however, functional validation is required to establish causal roles.

## Materials and methods

### Identification and characterization of *VpGYF* genes in the *V. planifolia*

The haplotype-resolved *V. planifolia* genome (CR0040 v2) and corresponding annotation files were downloaded from the Vanilla Genome Hub (https://vanilla-genome-hub.cirad.fr/). The Hidden Markov Model (HMM) profiles of the GYF domain (PF02213) and the GYF2 domain (PF14237) were obtained from Pfam (http://pfam.xfam.org/). Putative GYF-domain proteins were identified by searching for the vanilla proteome using the HMMER module implemented in TBtools-II (E-value < 1×10^-5^) (Chen et al., 2020). Redundant hits and truncated sequences were removed, and the remaining candidates were further verified for the presence of the PF02213 or PF14237 domain using InterProScan (https://www.ebi.ac.uk/interpro/), (Jones et al., 2014) SMART (http://smart.embl-heidelberg.de/), and GenomeNet (https://www.genome.jp/). Candidate genes were named *VpGYF1*–*VpGYF22* according to their chromosomal positions and *Arabidopsis* homologs.

For each VpGYF protein, the molecular weight, theoretical pI, instability index, aliphatic index, and grand average of hydropathicity (GRAVY) were calculated using ProtParam (https://web.expasy.org/protparam/). Subcellular localization was predicted with CELLO (http://cello.life.nctu.edu.tw/) (Yu et al., 2014). Additional sequence features of the VpGYF proteins are provided in **Supplementary Table 1**; the presence and positional information of GYF domains were determined using Pfam/InterPro annotations (**Supplementary Table 2**).

### Phylogenetic analysis, motif identification, domain organization, and gene structure

Full-length amino-acid sequences of VpGYF proteins and GYF proteins from *Arabidopsis thaliana*, *Oryza sativa*, and *Solanum lycopersicum* were retrieved from the NCBI Genome and Phytozome databases. Multiple sequence alignment was performed in MEGA X using MUSCLE with default parameters. Phylogenetic trees were constructed using the maximum-likelihood method with 1, 000 bootstrap replicates and visualized with iTOL v6 (https://itol.embl.de/) (Kumar et al., 2018; Letunic and Bork, 2021).

Conserved motifs in VpGYF proteins were identified using MEME v5.5.5 (https://meme-suite.org) with the following parameters: maximum number of motifs = 10, motif width = 6–50 amino acids, and E-value < 1×10^-5^. Conserved domains, including the GYF and associated auxiliary domains (e.g., SET, *SDG2*_C, Plus-3, SWIB, zf-CCCH), were annotated using InterProScan and SMART. Exon–intron organization and UTRs were obtained from the genome annotation (GFF3) and visualized together with motifs and domains in TBtools-II (Chen et al., 2020).

### Chromosomal localization, intra-genomic collinearity, inter-genomic synteny, and Ka/Ks analysis

Genomic coordinates of *VpGYF* genes were extracted from the CR0040 v2 annotation and mapped onto the H1 and H2 chromosomes (the two haplotypes of the V. planifolia genome) using TBtools-II. Intra-genomic collinearity blocks and homologous relationships between H1 and H2 were identified using the MCScanX toolkit in TBtools-II with default parameters. Pairwise nonsynonymous (Ka) and synonymous (Ks) substitution rates and Ka/Ks (ω) ratios between duplicated *VpGYF* genes were estimated with the KaKs_Calculator module (Nei–Gojobori method) in TBtools-II. The statistical significance of the deviation of ω from 1 was evaluated using the Z-test implemented in PAML’s codeml, with *P* < 0.05 considered significant (Yang, 2007).

To assess conservation of *VpGYF* loci across species, inter-genomic synteny was analyzed between *V. planifolia* and *A. thaliana*, *O. sativa*, *Phalaenopsis equestris*, *Dendrobium catenatum*, and *Apostasia shenzhenica*. Reciprocal BLASTP searches were performed (E-value < 1×10^-5^), and collinear blocks were detected with MCScanX in TBtools-II. Inter-species syntenic links containing *VpGYF* genes and their orthologous loci were visualized using the Advanced Circos function in TBtools-II (Chen et al., 2020).

### Promoter cis-element analysis of VpGYF genes

For each *VpGYF* gene, a 2, 000-bp sequence upstream of the translation start codon was extracted from the *V. planifolia* genome and submitted to PlantCARE (https://bioinformatics.psb.ugent.be/webtools/plantcare/html/) for prediction of cis-regulatory elements. Identified motifs were manually grouped into functional categories (hormone-responsive, stress/defense-related, light-responsive, transcription-factor binding sites such as MYB, MYC/bHLH, and WRKY) (Lescot, 2002). The number and distribution of cis-elements in each promoter were plotted in TBtools-II (Chen et al., 2020).

### Protein-protein interaction network and functional enrichment of the At*EXA1* module

Because several *VpGYF* genes are orthologous to *A. thaliana EXA1*, the *EXA1*-centered protein–protein interaction (PPI) network was analyzed in STRING v12 (https://string-db.org/) using default settings and a medium confidence score (0.4). The resulting interaction network was visualized in STRING, and proteins with annotated roles in viral processes were highlighted (Szklarczyk et al., 2023). Co-expression information for *EXA1* and its partners in *A. thaliana* and other organisms was obtained from the STRING co-expression matrices. Gene Ontology (GO) enrichment analysis of *EXA1*-network genes was performed using ShinyGO (http://bioinformatics.sdstate.edu/go/) with FDR < 0.05 as the significance threshold, and enriched biological process, molecular function, and cellular component terms were summarized (Ge et al., 2020).

### Transcriptome analysis of *VpGYF* genes in fruit development

Transcriptomic data for *V. planifolia* fruit development were retrieved from the NCBI Sequence Read Archive under BioProject PRJNA974693. Briefly, this dataset comprises RNA-seq libraries generated from fruits of two *V. planifolia* accessions with contrasting vanillin content, sampled at three developmental stages (6, 7, and 8 months after pollination) and two tissue types: outer green tissue (epicarp and residual mesocarp) and inner white tissue (mesocarp, endocarp, placenta, and seeds). Normalized expression values (TPM) for *VpGYF* genes were extracted from the published expression matrices. For visualization, TPM values were transformed as log2(TPM + 1) and then z-score normalized per gene (mean = 0, SD = 1 across samples) to enable comparison of expression patterns across tissues, stages, and accessions. Hierarchical clustering was performed using Euclidean distance with complete linkage, and heatmaps were generated using the pheatmap package in R.

### Virus inoculation and symptom evaluation

*Vanilla planifolia* ‘AG3’ plants were obtained from a single commercial nursery to ensure genetic consistency and minimize environmental variability. Prior to the experiment, plants were determined to be virus free using ELISA strip tests (Agdia, Inc, Indiana, USA). These immunostrip assays were used only for qualitative detection of CymMV presence or absence and not for quantitative coat-protein measurements. For pathogen challenge experiments, plants were maintained in a controlled greenhouse environment at 24–28 °C with relative humidity of 70–80% and a 16-hour photoperiod supplied by supplemental lighting. Plants were randomly assigned to two treatment groups: one set was mechanically inoculated with CymMV, while the other served as non-infected controls. Each treatment initially consisted of twelve plants arranged across four sampling blocks (P1–P4). Sampling blocks were used to distribute plants spatially within the experiment and to minimize positional effects during tissue collection. For RT-qPCR analysis at each time point, three plants were randomly selected from the control treatment and three plants were randomly selected from the CymMV-inoculated treatment for RNA extraction and downstream expression analysis. CymMV inoculum was prepared by homogenizing fresh or frozen CymMV-infected vanilla leaves in 0.02 M phosphate buffer (pH 7.0) at a ratio of 1 g tissue per 5 mL buffer. Silicon carbide powder was lightly dusted onto the adaxial leaf surface to enhance infection efficiency, and the inoculum was mechanically applied using sterile cotton swabs to 2–3 leaves immediately below the apical shoot. Control plants underwent identical treatment using virus-free phosphate buffer. Plants were monitored for symptom development, and systematic sampling commenced one-month post-inoculation. The youngest fully expanded leaf above the inoculation site was collected weekly for nine consecutive weeks.

### RNA extraction and qRT-PCR analysis

Harvested leaf tissue was immediately flash-frozen in liquid nitrogen, ground to a fine powder using a mortar and pestle and stored at −80 °C until processing. Total RNA was extracted using the RNeasy Plant Mini Kit (Qiagen, Germany) according to the manufacturer’s protocol. RNA concentration and purity were assessed using a NanoDrop spectrophotometer (Thermo Fisher Scientific, USA), with A260/A280 ratios between 1.8 and 2.1 considered acceptable. RNA integrity was verified by 1.5% agarose gel electrophoresis and Qubit fluorometric quantification (Thermo Fisher Scientific, USA); only samples with RNA Integrity Number (RIN) > 7.0 were used for downstream analysis. Complementary DNA (cDNA) was synthesized from 1 μg total RNA using amfiRivert Sensi cDNA Synthesis Master Mix (GenDEPOT, USA) in a 20 μL reaction volume under the following thermal conditions: 25 °C for 5 min (annealing), 37–55 °C for 30–60 min (extension), and 85 °C for 1 min (heat inactivation). Synthesized cDNA was diluted 1:10 with nuclease-free water and stored at −20 °C.

Expression levels of *VpGYF* genes were quantified using a QuantStudio 6 Real-Time PCR System (Thermo Fisher Scientific, USA) with PowerUp SYBR Green Master Mix (Applied Biosystems, USA). Gene-specific primers targeting all 11 *VpGYF* homologous pairs were designed using Primer3 software (**Supplementary Table 3**). *ACTIN* served as the internal reference gene. Its expression stability under CymMV infection was verified across all samples by examining Ct variance among control and infected plants, confirming minimal variation suitable for normalization 3/18/2026 6:51:00 AM. Each 10 μL reaction contained 5 μL of 2X SYBR Green Master Mix, 0.5 μL each of forward and reverse primers (10 μM), 1 μL diluted cDNA template, and 3 μL nuclease-free water. Thermal cycling conditions consisted of initial denaturation at 95 °C for 10 min, followed by 40 cycles of 95 °C for 15 s, 60 °C for 30 s, and 72 °C for 30 s. Melt curve analysis was performed after amplification to confirm primer specificity. All reactions were performed in technical triplicate, and no-template controls were included to monitor contamination. Primer amplification efficiency was validated using five-point standard dilution curves, with efficiencies between 90% and 110% considered acceptable.

Cycle threshold (Ct) values were exported from QuantStudio 6 software. Each biological replicate represented an independent plant (n = 3 plants per treatment per time point). ΔCt was calculated per plant as Ct(target) − Ct(ACTIN). For each gene and week, ΔΔCt was calculated as ΔCt(challenged) − mean ΔCt(control) using time-matched control plants as the calibrator. Expression changes were reported as log2 fold change (log2FC = −ΔΔCt) and summarized as mean ± standard error (SE) across biological replicates. Differences between challenged and control plants at each time point were assessed using Student’s t-test (P < 0.05).

## Results

### Identification and evolutionary relationships of *GYF* genes in *V. planifolia*

**Figure 1:**
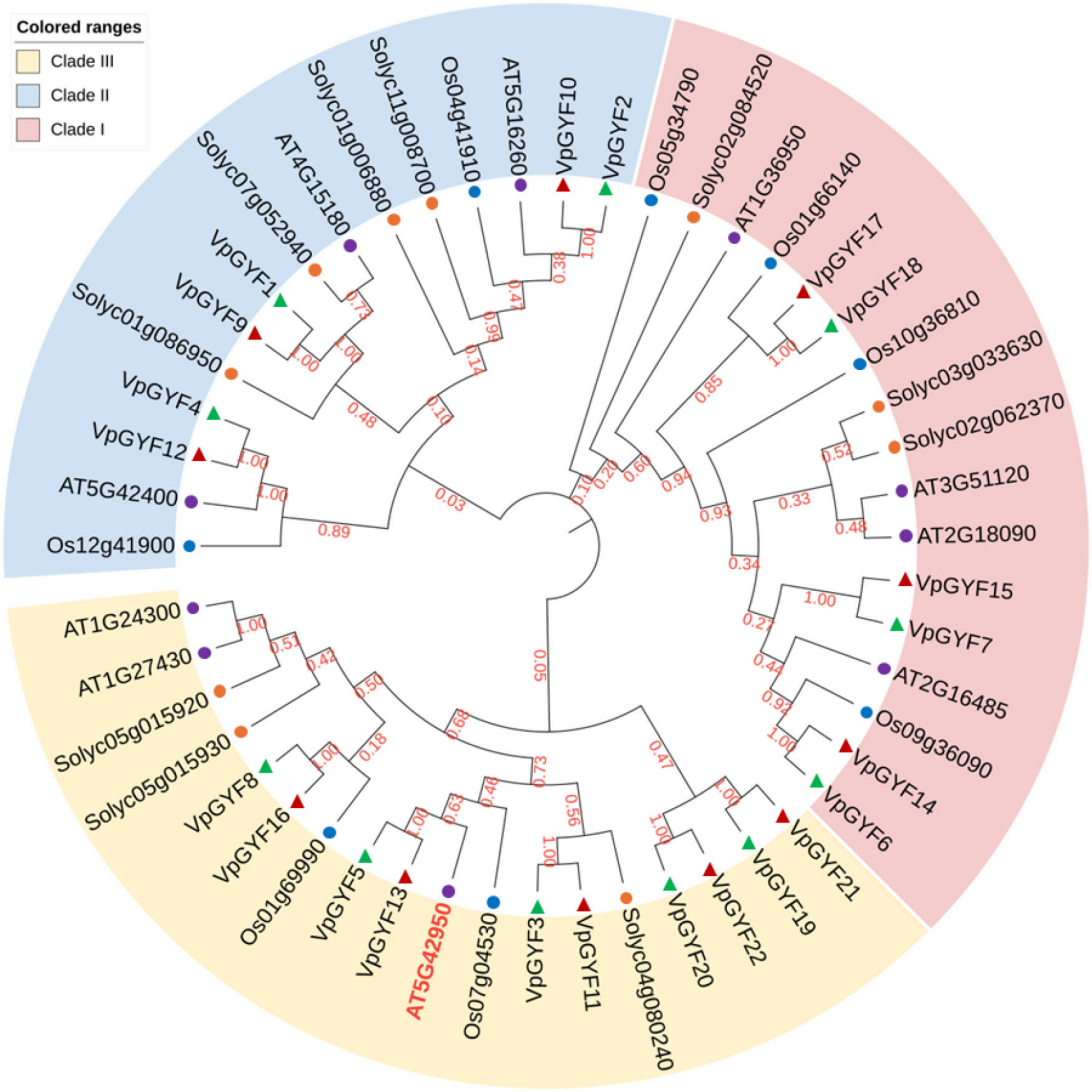
Phylogenetic relationships of GYF proteins from *V. planifolia*, *A. thaliana*, *O. sativa* and *S. lycopersicum*. The full-length amino acid sequences of 22 VpGYF proteins, together with representative GYF proteins from *Arabidopsis thaliana* (At; purple circles), *Oryza sativa* (Os; blue circles) and *Solanum lycopersicum* (Solyc; orange circles), were used to construct a circular phylogenetic tree using the maximum-likelihood method with 1, 000 bootstrap replicates. Vanilla proteins encoded by the H1 and H2 haplotypes are indicated by green and red triangles, respectively. The tree is partitioned into three major clades (Clades I–III), which are marked by the colored sectors in the outer ring. Bootstrap support values are shown in red at the corresponding internal nodes.

We scanned all predicted proteins in the haplotype-resolved *V. planifolia* (haplotype-phased assembly CR0040 v2) reference genome for the conserved GYF domain (Pfam PF02213) and GYF2 domain (Pfam PF14237), complemented by BLASTP searches using *Arabidopsis* GYF proteins as queries (Piet et al., 2022). This analysis identified 11 *GYF* loci, each represented by two phased allelic copies (H1 and H2), resulting in 22 annotated gene models, designated *VpGYF1*–*VpGYF22* (**Table 1**). These loci are distributed unevenly across H1/H2 chromosome pairs and occur predominantly as homologous H1/H2 pairs (**Figure 1**), consistent with the phased diploid genome structure. A maximum-likelihood phylogenetic tree constructed from full-length amino acid sequences of *V. planifolia*, *A. thaliana*, *Oryza sativa*, and *Solanum lycopersicum* GYF proteins resolved three well-supported clades (**Figure 1**). Each clade contains representatives from all four species, indicating the conservative nature of GYF across angiosperms.

**Table 1:** Characterization of *VpGYF* genes in *V. planifolia*.

Nomenclature	Gene ID	Chr	Start	End	Strand	CDS length	aa length	Homolog in Arabidopsis	
VpGYF1	VPCR0040H1_01G018200	H1_01	190447729	190460861	+	7017	2338	AT4G15180	*ATXR3*
VpGYF2	VPCR0040H1_05G011030	H1_05	25893041	25921050	–	1485	494	AT5G16260	*ELF9*
VpGYF3	VPCR0040H1_09G013090	H1_09	80959708	80974733	-	5241	1746	AT5G42950	*EXA1*
VpGYF4	VPCR0040H1_10G015910	H1_10	117450608	117465451	–	3777	1258	AT5G42400	*ATXR7*
VpGYF5	VPCR0040H1_13G006070	H1_13	58750396	58763509	+	5673	1890	AT5G42950	*EXA1*
VpGYF6	VPCR0040H1_15G002400	H1_15	4189879	4209895	–	5493	1830	AT2G16485	*NERD*
VpGYF7	VPCR0040H1_16G000240	H1_16	538185	578575	+	4392	1463	AT2G16485	*NERD*
VpGYF8	VPCR0040H1_16G011600	H1_16	52809052	52818487	–	4971	1656	AT1G24300	F3I6.24
VpGYF9	VPCR0040H2_01G024290	H2_01	173342714	173355863	+	7017	2338	AT4G15180	*ATXR3*
VpGYF10	VPCR0040H2_05G012040	H2_05	27343034	27371043	–	1485	494	AT5G16260	*ELF9*
VpGYF11	VPCR0040H2_09G020020	H2_09	181668863	181683901	-	5268	1755	AT5G42950	*EXA1*
VpGYF12	VPCR0040H2_10G012540	H2_10	34841946	34856809	–	3729	1242	AT5G42400	*ATXR7*
VpGYF13	VPCR0040H2_13G006130	H2_13	42839096	42852224	+	5667	1888	AT5G42950	*EXA1*
VpGYF14	VPCR0040H2_15G002570	H2_15	4074380	4094372	–	5493	1830	AT2G16485	*NERD*
VpGYF15	VPCR0040H2_16G000480	H2_16	931437	971485	-	4383	1460	AT2G16485	*NERD*
VpGYF16	VPCR0040H2_16G006880	H2_16	76724554	76734000	–	4980	1659	AT1G24300	F3I6.24
VpGYF17	VPCR0040H2_16G011320	H2_16	84591598	84629452	+	2397	798	AT5G63700	MBK5.18
VpGYF18	VPCR0040H1_16G016220	H1_16	60759877	60797731	+	2397	798	AT5G63700	MBK5.18
VpGYF19	VPCR0040H1_15G007110	H1_15	12345818	12363924	-	1494	497	AT5G01590	TIC56
VpGYF20	VPCR0040H1_07G011340	H1_07	69672803	69711249	–	7830	2609	AT2G26890	GRV2
VpGYF21	VPCR0040H2_15G007020	H2_15	11816798	11834837	-	1494	497	AT5G01590	TIC56
VpGYF22	VPCR0040H2_07G017390	H2_07	65266533	65304979	–	7830	2609	AT2G26890	GRV2

Clade I (blue) encompasses chromatin-modification and RNA-processing proteins. The vanilla homologous pair *VpGYF1/VpGYF9* clustered with *Arabidopsis ATXR3* (AT4G15180) and tomato Solyc07g052940 (bootstrap = 1.00), corresponding to the H3K4 histone methyltransferase lineage. *VpGYF4/VpGYF12* grouped with *Arabidopsis ATXR7* (AT5G42400) and rice Os12g41900, the *SDG25* histone methyltransferase subfamily. A third subclade contained *VpGYF2/VpGYF10*, with Arabidopsis *ELF9* (AT5G16260), rice Os04g41910, and tomato Solyc11g008700, all of which are RNA-binding/splicing factors. Clade II (pink) displayed the greatest domain diversity. The *NERD*-like subclade (bootstrap = 0.92-1.00) included *VpGYF6/VpGYF14* and *VpGYF7/VpGYF15*, which clustered with *Arabidopsis NERD* (AT2G16485) and rice orthologs Os09g36090 and Os10g36810, proteins characterized by complex domain architectures combining GYF with SWIB, PHD, and Plus-3 domains. *VpGYF17/VpGYF18* formed a sister group with rice Os01g66140, representing a lineage divergent from canonical *NERD*. *Arabidopsis* AT1G36950 and tomato Solyc02g084520 occupied basal positions within Clade II, suggesting ancestral GYF configurations. Clade III (yellow) contains *EXA1*-like susceptibility factors. The *EXA1* subclade (bootstrap = 0.73) included *VpGYF3/VpGYF11* and *VpGYF5/VpGYF13* clustering with *Arabidopsis EXA1* (AT5G42950), rice Os07g04530, and tomato Solyc04g080240. Vanilla thus harbors four *EXA1*-like genes, compared with a single copy in *Arabidopsis*, either orchid-specific expansion or differential retention following whole-genome duplication. *VpGYF8/VpGYF16* grouped with *Arabidopsis* F3I6.24 (AT1G24300, AT1G27430), rice Os01g69990, and tomato Solyc05g015920/Solyc05g015930 in a related subclade. The remaining proteins (*VpGYF19/VpGYF21* and *VpGYF20/VpGYF22*) formed a well-supported sister group (bootstrap = 0.47-1.00) at the base of Clade III, possibly vanilla-specific expansions. All vanilla homologous pairs exhibited bootstrap values of 1.00, confirming their origin from whole-genome duplication.

### *VpGYF* genes exhibit conserved motifs, domain organization, and diverse gene structures

**Figure 2:**
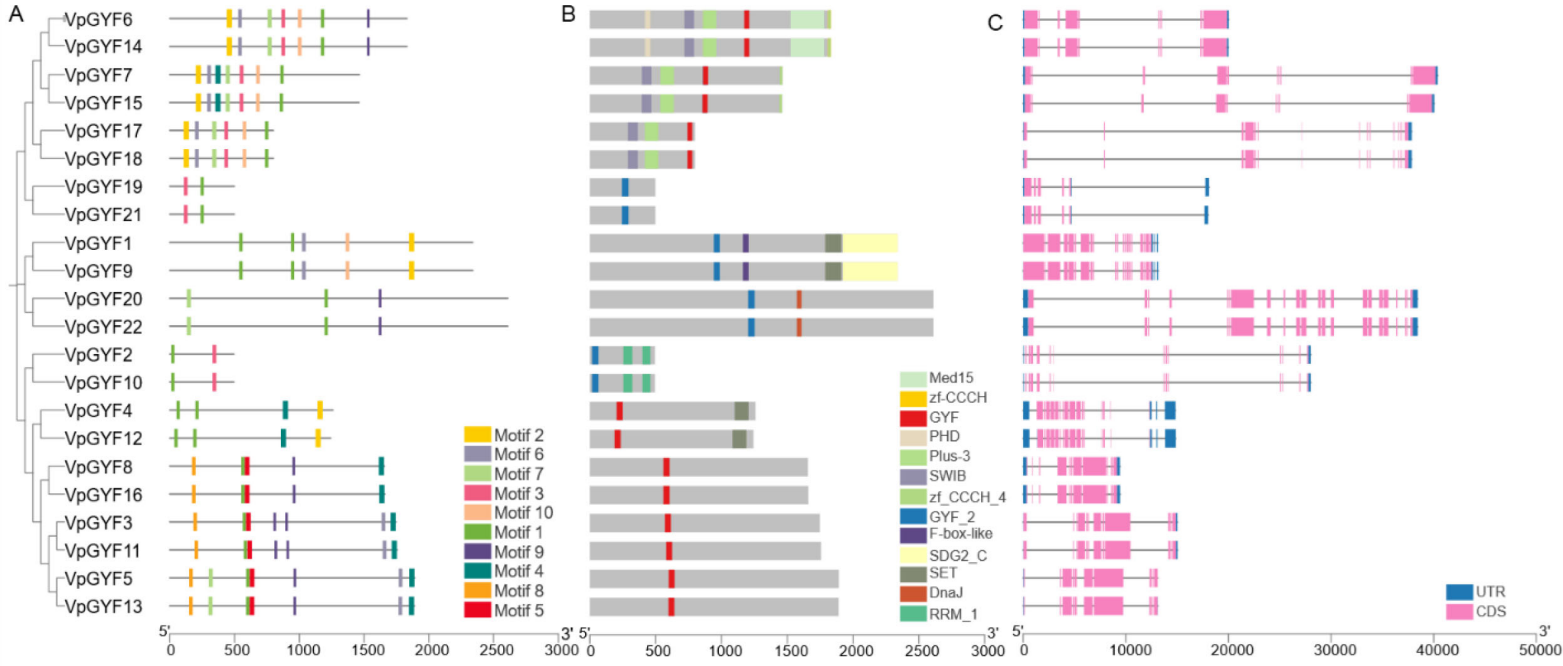
Phylogenetic relationships, conserved motifs, domain architectures and gene structures of VpGYF proteins. **(A)** Phylogenetic tree of the 22 VpGYF proteins and distribution of 10 conserved motifs identified by MEME. Colored boxes (Motifs 1–10) indicate different motifs, and their positions reflect their relative locations within each protein. **(B)** Schematic representation of conserved domains in VpGYF proteins. Grey bars represent full-length proteins, and colored boxes denote annotated domains (GYF, GYF_2, F-box-like, *SDG2*_C, SET, RRM_1, Plus-3, SWIB, Med15, zf-CCCH, PHD, and zf-CCCH_4). **(C)** Gene structures of *VpGYF* genes. Pink and blue boxes indicate coding sequences (CDS) and untranslated regions (UTRs), respectively, and black horizontal lines represent introns. Scales at the bottom indicate protein length (aa) or genomic length (bp).

MEME analysis identified 10 conserved motifs among the 22 VpGYF proteins. Motif 3 overlaps the canonical GYF domain and occurs in nearly all family members, centrally positioned in the single-domain proteins (VpGYF3, VpGYF5, VpGYF11, VpGYF13). Motifs 1, 2, and 4 flank motif 3 in most proteins with clade-specific combinations. Homologous pairs share nearly identical motif patterns, consistent with Haplotype 1 (H1) and Haplotype 2 (H2) genome duplication. Domain annotations revealed structural diversification beyond the core GYF/GYF_2 module. VpGYF1 and VpGYF9 fuse GYF_2 to C-terminal SET and *SDG2*_C domains, the hallmark of H3K4 methyltransferases. VpGYF6, VpGYF7, VpGYF14, and VpGYF15 combine GYF with F-box-like, Plus-3, SWIB, PHD, and zf-CCCH_4 domains, implicating E3-ligase and chromatin-associated functions. VpGYF8 and VpGYF16 associate GYF with zf-CCCH domains. By contrast, VpGYF3, VpGYF5, VpGYF11, and VpGYF13 are simple single-GYF scaffolds. This domain architecture diversity integrates the conserved GYF peptide-binding module into chromatin, transcriptional, and post-transcriptional regulatory contexts (**Figure 2**, **Supplementary Figure 1**).

Gene structure varies substantially across the family. Exon number ranges from 10 to 22 (mean ≈13.8), and gene length spans 9.6 kb (*VpGYF8*, *VpGYF16*) to 40.4 kb (*VpGYF7*, *VpGYF15*). Homologous H1/H2 pairs display strikingly similar exon–intron patterns and UTR arrangements. The coexistence of compact genes (10–11 exons within <15 kb) alongside intron-dense loci (up to 22 exons spanning ~40 kb) indicates that both intron gain and intron loss shaped VpGYF family evolution (**Figure 2**).

### *VpGYF* genes show homologous chromosomal distribution and orchid-biased synteny

**Figure 3:**
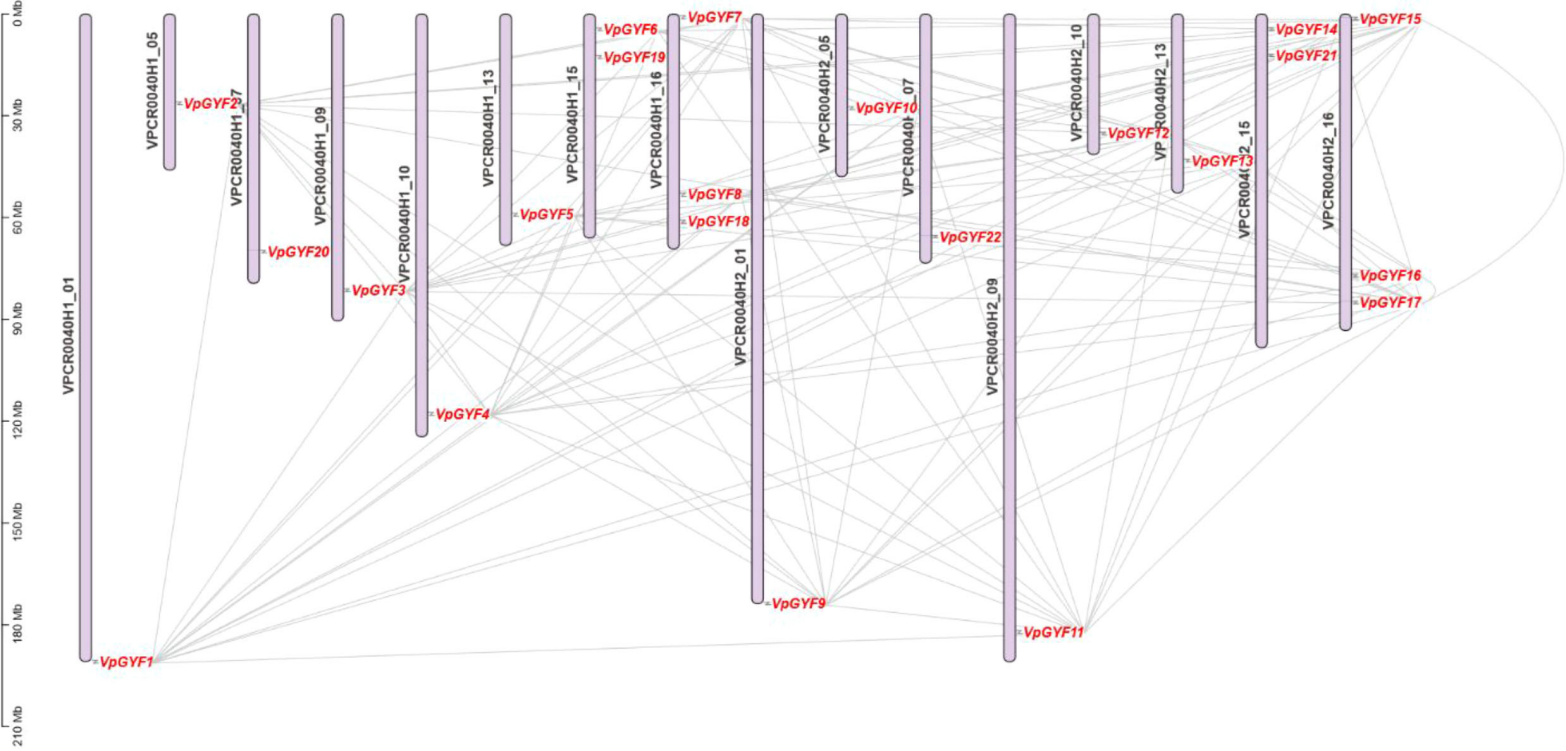
Chromosomal distribution and syntenic relationships of *VpGYF* genes in the *V. planifolia* genome. 22 *VpGYF* genes are mapped onto the haplotype-resolved chromosomes of the H1 and H2 haplotypes, shown as vertical bars with chromosome IDs indicated at the top. Gene positions are marked in red along each chromosome, and the scale on the left is given in megabases (Mb). Grey connecting lines represent collinear blocks between H1 and H2 chromosomes, illustrating homologous relationships and conserved synteny among *VpGYF* loci.

The 22 *VpGYF* genes distribute across the haplotype-resolved genome as H1/H2 homologous pairs (**Figure 3**; **Table 1**). Most chromosomes carry a single *VpGYF* locus. Exceptions include H1_15 and H2_15, which harbor two-gene clusters (*VpGYF6*, *VpGYF19*) and (*VpGYF14*, *VpGYF21*), and H1_16 and H2_16, which contain three-gene clusters (*VpGYF7*, *VpGYF8*, *VpGYF18*) and (*VpGYF15*-*VpGYF17*). Ka/Ks analysis of 103 duplicate pairs (**Supplementary Table 2**) yielded mean and median values of 0.74 and 0.76, purifying selection predominates. Eighteen pairs (including *VpGYF3*-*VpGYF11*, *VpGYF5*-*VpGYF13*, *VpGYF8*-*VpGYF16*) exhibited Ka/Ks < 0.5 (strongly constrained). Four pairs (*VpGYF1*-*VpGYF4*, *VpGYF1*-*VpGYF12*, *VpGYF4*-*VpGYF9*, *VpGYF9*-*VpGYF12*) showed Ka/Ks ≈ 1.27, candidates for diversifying selection (**Supplementary Table 2**).

**Figure 4:**
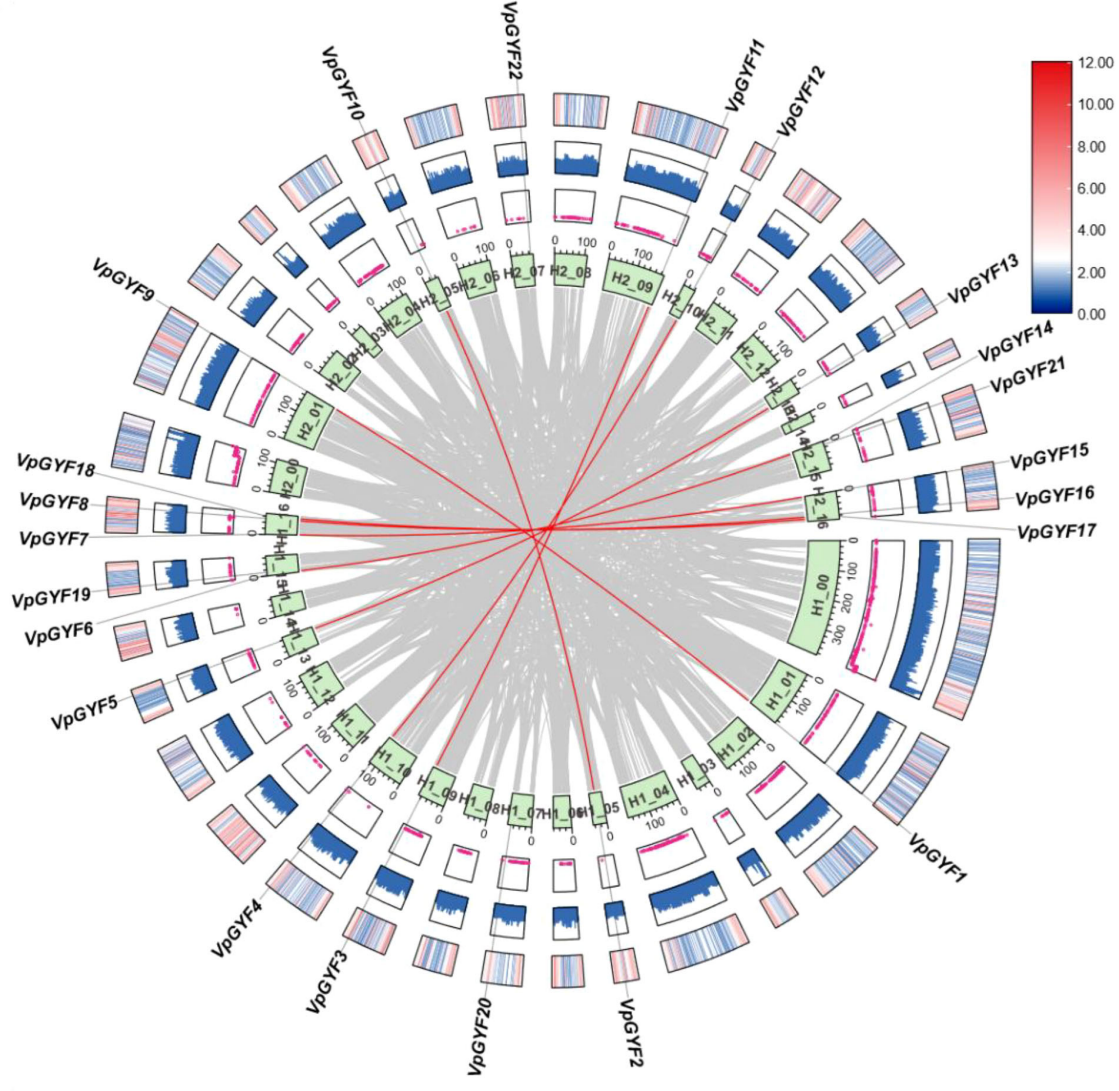
Intra-genomic synteny and genomic features surrounding *VpGYF* genes in *V.planifolia.* Grey ribbons in the center represent genome-wide collinear blocks, and the red line represents the pairwise replication of the *VpGYF* loci to their chromosomal positions. The green ring shows chromosomes from the H1 and H2 haplotypes, labeled accordingly. From the inside to the outside, the subsequent tracks display Nratio values (magenta lines), GC content (blue bar plots) and gene-density distribution (heatmap; blue to red scale). Gene names (*VpGYF1*-*VpGYF17*) are indicated around the outer circle.

Intra-genomic collinearity analysis revealed 11 homologous pairs linked by dense H1 and H2 syntenic blocks (**Figures 3**, **4**). Tandem arrays were not detected and whole-genome duplication followed by differential homeolog retention, rather than local duplication, primarily shaped like the *VpGYF* repertoire. Circos visualization confirmed that *VpGYF* loci reside in low-N regions with typical GC content and moderate-to-high surrounding gene density, indicating well-assembled, gene-rich chromosomal contexts (**Figure 4**).

**Figure 5:**
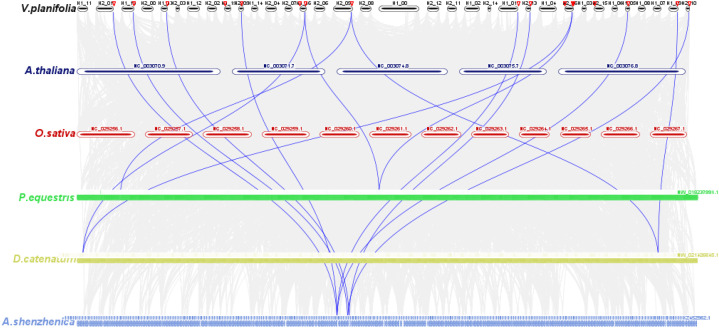
Inter-genomic synteny analysis of *VpGYF* loci across plant genomes. Chromosomes of *V. planifolia* (top track, H1/H2 haplotypes), *Arabidopsis thaliana* (*A. thaliana*), *Oryza sativa* (*O. sativa*), *Phalaenopsis equestris* (*P. equestris*), *Dendrobium catenatum* (*D. catenatum*) and *Apostasia shenzhenica* (*A. shenzhenica*) are shown as colored bars. Grey background lines represent genome-wide collinear blocks, whereas blue curves highlight syntenic segments containing *VpGYF* genes and their orthologous loci in the other species, illustrating conserved macrosynteny of VpGYF-containing regions primarily within Orchidaceae, whereas no detectable collinearity was observed with *A. thaliana* or *O. sativa* under the parameters used.

Inter-genomic synteny analysis across six species revealed no detectable collinearity between *VpGYF*-containing segments and *A. thaliana* or *O. sativa*, but clear macrosystemic links within Orchidaceae: four syntenic blocks with *P. equestris*, two with *D. catenatum*, and eight with the basal orchid *A. shenzhenica* (**Figure 5**). The chromosomal neighborhoods harboring *VpGYF* genes have been preferentially conserved within orchids, while broader collinearity with eudicot and grass genomes eroded, ancient origin coupled with lineage-specific evolution.

### *VpGYF* genes possess diverse regulatory elements in their promoter regions

**Figure 6:**
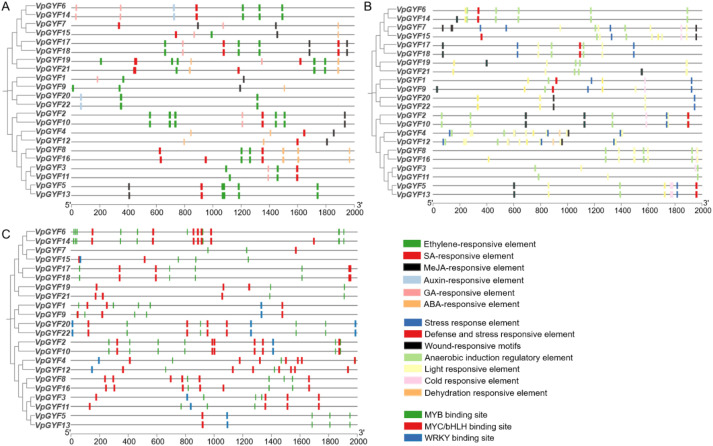
Cis-element analysis of *VpGYF* gene promoters in *V. planifolia*. Cis-regulatory elements were predicted in the 2, 000-bp upstream regions of *VpGYF* genes. Colored boxes along each horizontal line indicate individual cis-elements and their relative positions, and the phylogenetic tree on the left shows relationships among *VpGYF* genes. **(A)** Hormone-responsive elements, including SA-, MeJA-, ethylene-, GA-, ABA- and auxin-responsive motifs. **(B)** Environmental and developmental cis-elements, including anaerobic-induction, circadian-control, cold-responsive, defense/stress-responsive, light-responsive, meristem-regulatory, wound-responsive and dehydration-responsive motifs. **(C)** Predicted transcription factor binding sites, including WRKY, MYB and MYC/bHLH binding motifs.

Analysis of the 2, 000-bp upstream regions of all 22 *VpGYF* genes identified 518 *cis*-elements (22–44 per promoter) spanning hormone signaling, stress responses, and transcription-factor binding (**Figures 6A–C**). Hormone-responsive elements totaled 136: 32 SA-responsive, 26 MeJA-responsive, 43 ethylene-responsive, 21 ABA-responsive, 12 GA-responsive, and 2 auxin-responsive. Stress-related elements totaled 111, including anaerobic induction, cold/dehydration, defense/stress-responsive, and 18 wound-responsive motifs. Light- and meristem-related elements (79 in total) occurred in nearly all promoters.

Transcription-factor binding sites were highly enriched: 107 MYB, 74 MYC/bHLH, and 11 WRKY sites distributed across the family (4–23 per promoter; **Figure 6**). The four *EXA1* orthologs (*VpGYF3*, *VpGYF5*, *VpGYF11*, *VpGYF13*) harbor characteristic combinations of SA-, MeJA-, and ethylene-responsive elements alongside WRKY, MYB, and MYC/bHLH binding sites. *VpGYF5* and *VpGYF13* additionally contain wound- and defense-responsive motifs, promoter architectures consistent with inducible expression through immune and wound-signaling pathways. These cis-regulatory elements were identified through computational promoter analysis and therefore represent predicted regulatory motifs. Experimental validation using approaches such as electrophoretic mobility shift assays (EMSA) or chromatin immunoprecipitation (ChIP-qPCR) will be required to confirm transcription factor binding *in vivo*.

### VpGYF proteins display structural and functional diversity

VpGYF proteins range from 494 to 2, 609 amino acids (55–267 kDa), are predominantly acidic and hydrophilic (GRAVY values -0.08 to -0.82), and mostly show instability indices >40, typical of regulatory proteins (**Supplementary Table 1**). VpGYF19/VpGYF21 are exceptions, with the lowest instability indices (34.83). The GYF domain (PF02213) is present in VpGYF1-VpGYF18, typically as a single internal module (e.g., residues 571–605 in VpGYF3, 598–635 in VpGYF13). VpGYF4 and VpGYF12 contain two GYF-like segments, specialized scaffolds for multiprotein complexes. The GYF2 domain (PF14237) occurs in 14 members; VpGYF19-VpGYF22 contain only this domain (**Supplementary Table 1**). Subcellular localization predictions indicate 19/22 proteins are nuclear; VpGYF2 and VpGYF10 are cytoplasmic; VpGYF19 and VpGYF21 are mitochondrial; VpGYF20 and VpGYF22 localize to multiple compartments.

To further explore the *VpGYF* family in vanilla, we investigated the Arabidopsis *EXA1* protein and its interaction network. The *Arabidopsis EXA1* interaction network, corresponding to VpGYF3, VpGYF5, VpGYF11, and VpGYF13, centers on translation initiation factors (eIF4E/eIF(iso)4E, eIF4G/eIF(iso)4G), cap-binding proteins, and RNA helicases (**Supplementary Figure 2**). Several partners (highlighted in red) have documented roles in virus infection or antiviral defense. Gene Ontology (GO) enrichment of the *EXA1* network shows over-representation of translation initiation, mRNA/cap binding, ribonucleoprotein complexes, and RNA metabolic processes (**Supplementary Figures 1**, **2**). The *EXA1*-like VpGYF proteins thus operate at the interface of mRNA metabolism, translation, and host-virus interactions. It should be noted that these interactions are derived from STRING database predictions based on homologous interaction networks and therefore represent putative associations rather than experimentally validated interactions. Experimental validation using approaches such as yeast two-hybrid assays or co-immunoprecipitation will be required to confirm these predicted interactions.

### *VpGYF* expression correlates with vanillin accumulation capacity during fruit development

**Figure 7:**
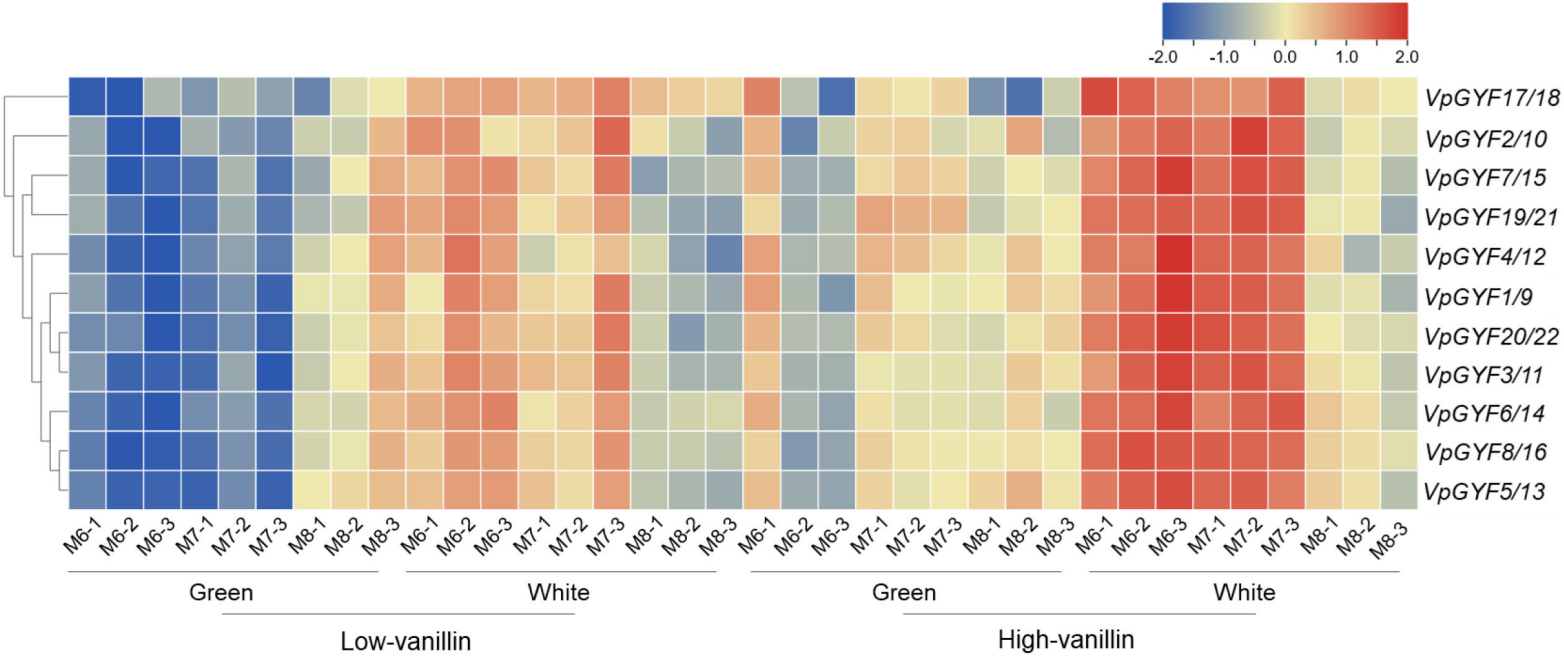
Expression profiles of *VpGYF* loci during vanilla fruit development. Heatmap showing z-score–normalized expression of 17 *VpGYF* loci in fruits of two accessions representing low-vanillin and high-vanillin, at months 6 to 8. Columns represent green (epicarp/outer mesocarp) and white (inner mesocarp/endocarp/placenta/seed) tissues of each accession and stage, and rows represent individual *VpGYF* loci clustered by hierarchical clustering. Colors indicate relative expression levels for each gene across all samples (blue, below the gene’s mean; white, near the mean; red, above the mean).

Transcriptome analyses of developing vanilla fruits revealed coordinated *VpGYF*expression patterns that distinguish the high-vanillin from low-vanillin accessions (**Figure 7**). The high-vanillin accession exhibited consistently higher *VpGYF* expression than low-vanillin accession across fruit tissues and developmental stages. In white tissue, inner mesocarp, endocarp, placenta, and seeds where glucovanillin accumulates, the z-scores of high-vanillin accession clustered at +1.1 to +1.5, with *VpGYF8/VpGYF16* at the upper range, while the low-vanilla accession remained moderately induced (+0.4 to +0.9). In green tissue (outer epicarp and residual mesocarp), the z-scores of high-vanillin accession showed mild repression (-0.2 to -0.6), while the low-vanilla accession displayed strong downregulation, *VpGYF8/VpGYF16* and *VpGYF6/VpGYF14* reached -1.7 to -1.8 at month 6 after pollination. Elevated *VpGYF* activity correlates with regulatory networks that favor efficient vanillin precursor production.

A clear spatial bias emerged: relative expression in white tissue exceeded green tissue at corresponding stages in both accessions, aligning with the concentration of vanillin biosynthetic machinery in the fruit inner tissue. Temporal dynamics differed between tissues. Green tissue expression increased from month 6 to 8, *VpGYF5/VpGYF13* in high-vanillin accession shifted from -0.4 to above 0, reflecting gradual activation as outer layers transition toward senescence. White tissue expression declined over time (e.g., *VpGYF4/VpGYF12* in low-vanillin accession decreased from strongly positive to near zero), indicating transient peaks during active glycovanillin accumulation followed by stabilization. Hierarchical clustering identified white-tissue-enriched pairs that resist this decline: *VpGYF8/VpGYF16 and VpGYF7/VpGYF15 maintain sustained induction in high-vanillin white tissue*, suggesting they may be candidate regulators of vanillin accumulation capacity.

### *VpGYF* transcriptional responses to CymMV infection parallel viral accumulation

**Figure 8:**
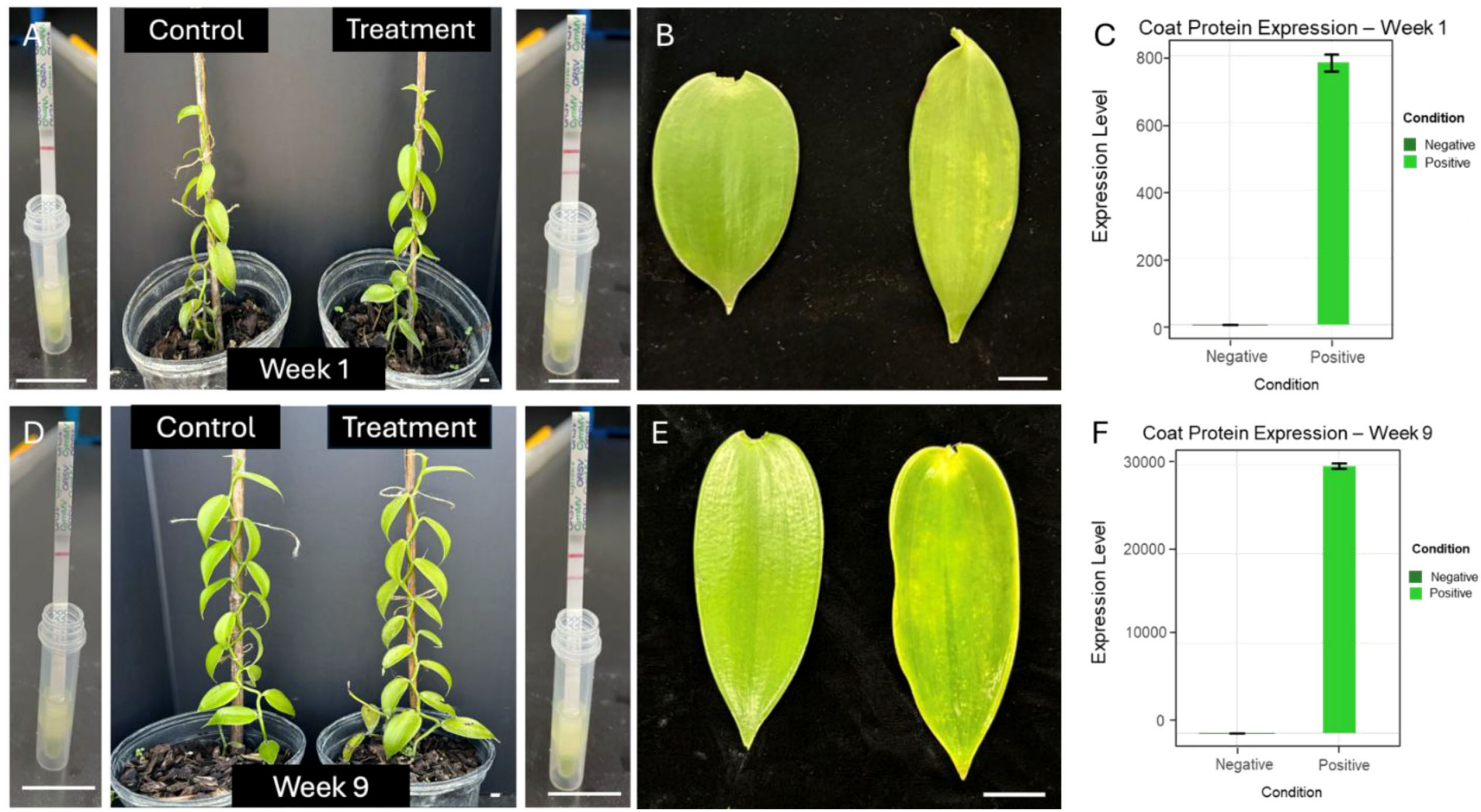
Phenotypic responses and CymMV coat-protein transcript accumulation in vanilla over a 9-week infection period. **(A)** Morphological comparison of control and CymMV-inoculated plants at Week 1. Early infection symptoms were subtle, with mild chlorosis beginning to appear in the treated plants. **(B)** Representative leaves from control (left) and infected (right) plants at Week 1, showing slight loss of pigmentation in the infected tissue. Scale bar = 1 cm. **(C)** Relative CymMV coat-protein transcript abundance at Week 1. Positive samples exhibited elevated coat-protein transcript accumulation relative to negative controls, consistent with early viral presence. Error bars represent standard deviation (n = 3). **(D)** Morphological comparison at Week 9. CymMV-infected plants displayed pronounced symptoms, including strong chlorosis, reduced vigor, and decreased overall growth relative to controls. **(E)** Representative leaves from control (left) and infected (right) plants at Week 9, showing severe yellowing and reduced leaf integrity. Scale bar = 1 cm. **(F)** Relative coat-protein transcript abundance at Week 9, demonstrating a substantial increase in viral accumulation in positive samples compared with negligible levels in controls. Error bars represent standard deviation (n = 3).

**Figure 9:**
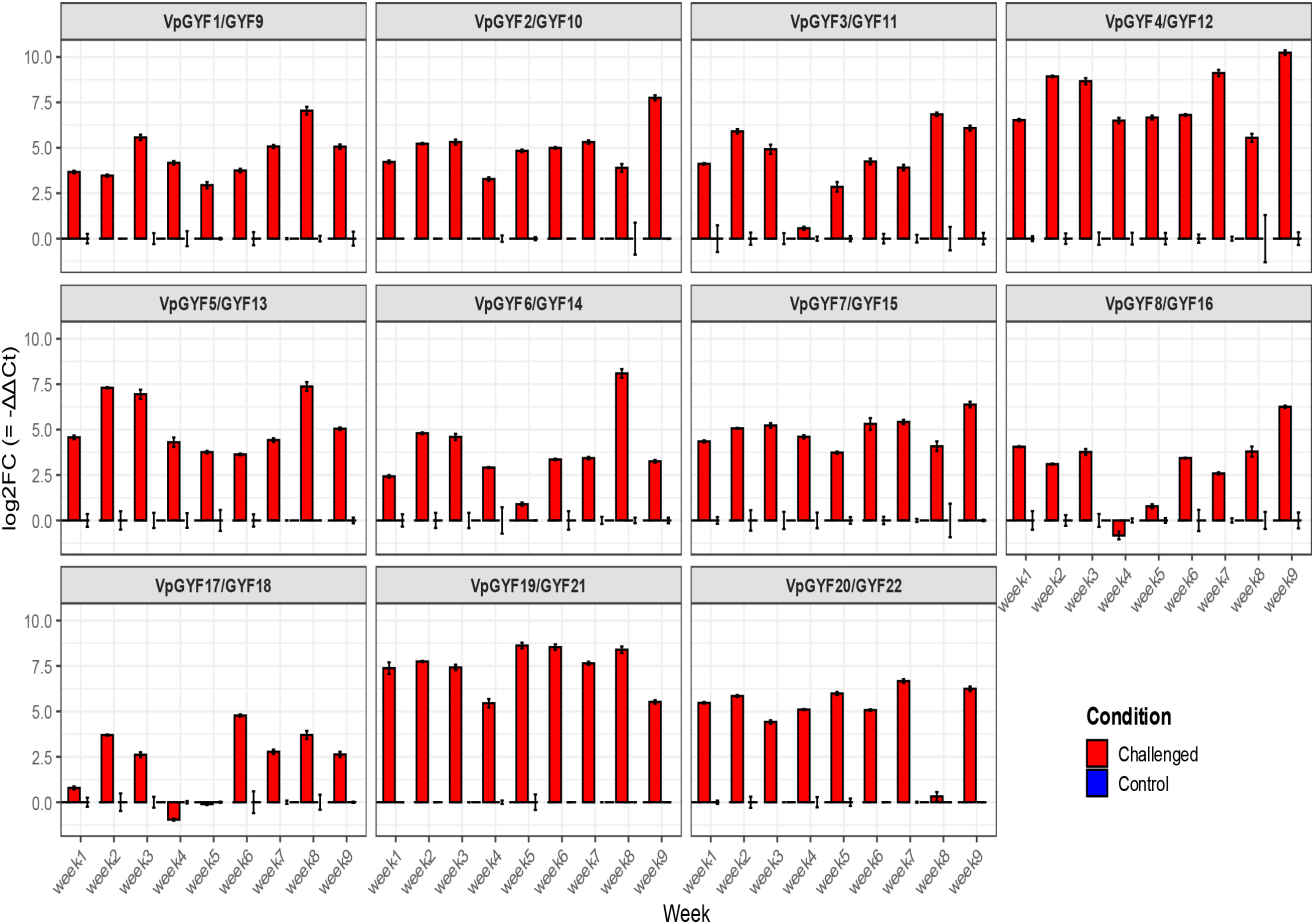
qRT-PCR expression profiles of VpGYF loci during CymMV infection. qRT-PCR was used to quantify transcript levels for 11 VpGYF homologous pairs in CymMV-challenged and time-matched control plants over a nine-week sampling period. Ct values were normalized to ACTIN (ΔCt), and infection-associated expression differences were calculated relative to time-matched controls (ΔΔCt) and reported as log2 fold change (log2FC = −ΔΔCt). Bars represent mean ± SE across three biological replicates (n = 3 plants per treatment per time point).

Successful mechanical inoculation with CymMV was confirmed by symptom progression and viral coat-protein transcript accumulation in vanilla leaves (**Figure 8**). Viral accumulation was estimated using the infected plants exhibited mild chlorosis relative to controls with corresponding leaf symptoms.~The 11 *VpGYF* homologous pairs were quantified by qRT-PCR weekly using ACTIN as the reference gene and time-matched control plants as the calibrator (**Figure 9**). Expression differences were calculated using the comparative Ct framework (ΔΔCt) and are presented as log2 fold change relative to control (log2FC = −ΔΔCt). Time-matched control plants were used as calibrators for ΔΔCt calculations, allowing infection-associated transcriptional changes to be evaluated across the nine-week sampling period. Across the time course, CymMV challenge elicited heterogeneous transcriptional responses among VpGYF loci, with both response magnitude and timing differing among family members. *VpGYF4/GYF12* and *VpGYF19/GYF21* exhibited the strongest and most sustained induction across multiple weeks, while *VpGYF6/GYF14* showed pronounced induction during mid-to-late sampling points. *VpGYF5/GYF13* and *VpGYF1/GYF9* displayed moderate but reproducible increases across several weeks, whereas *VpGYF8/GYF16* and *VpGYF17/GYF18* showed comparatively weaker or transient responses at specific time points. Overall, these results indicate that CymMV infection induces differential transcriptional responses among VpGYF loci rather than uniform activation of the entire gene family.

**Figure 10:**
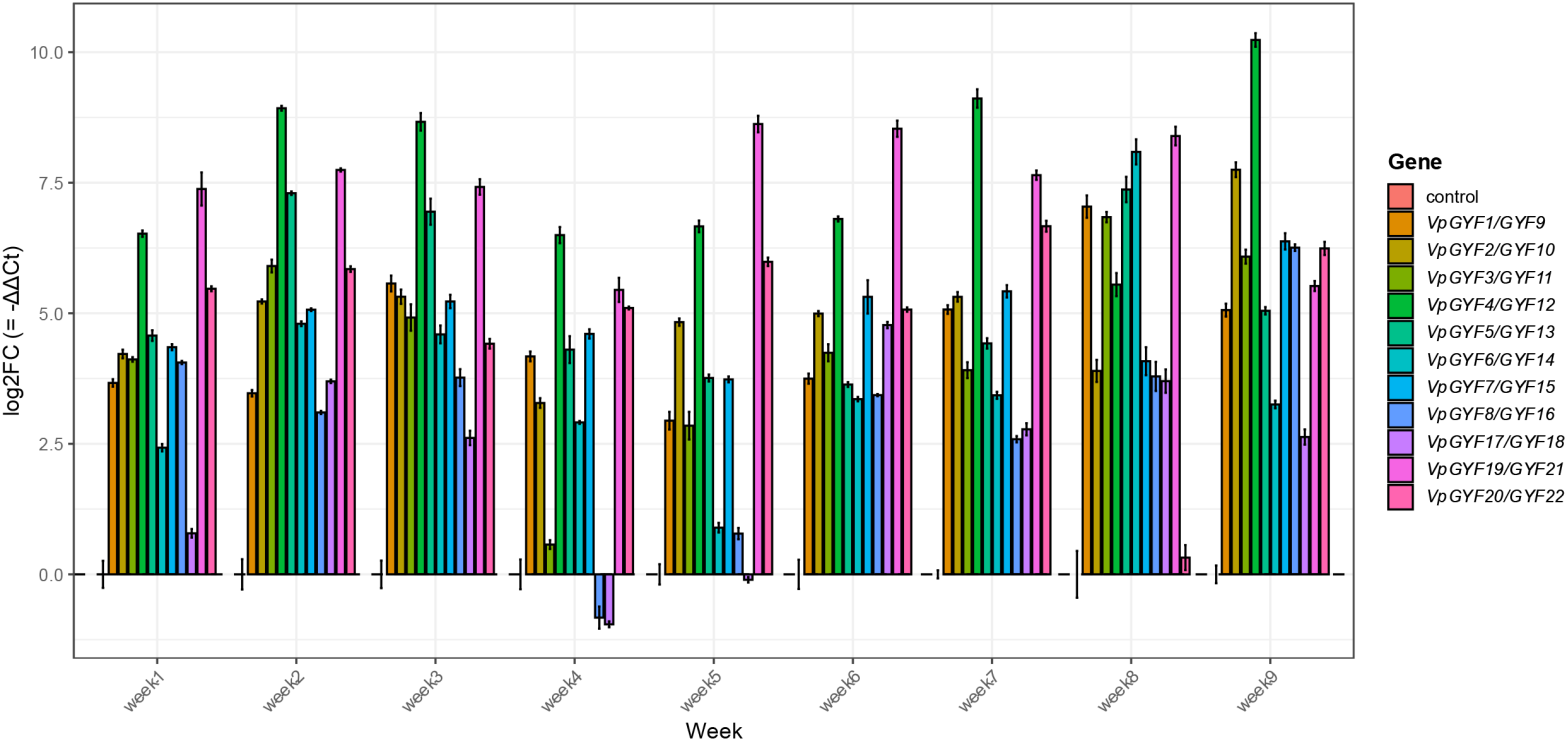
Consolidated comparison of CymMV-induced expression changes across the VpGYF family. The plot summarizes log2 fold change values (log2FC = −ΔΔCt) for all 11 VpGYF homologous pairs across the nine-week infection time course, calculated relative to time-matched controls. This consolidated view enables direct comparison of response magnitude and timing among VpGYF loci. Values represent mean ± SE across three biological replicates.

Consolidated visualization of log2FC values (**Figure 10**) enabled direct comparison of infection-associated response magnitudes across the *VpGYF* family. *VpGYF4/GYF12*, *VpGYF19/GYF21*, and *VpGYF6/GYF14* consistently displayed the highest log2FC values across multiple weeks, whereas other loci exhibited lower-magnitude or more transient expression changes over the infection time course.

## Discussion

### The *VpGYF* gene family: structural diversity and evolutionary conservation

This study constitutes the first genome-wide characterization of the GYF gene family in *Vanilla planifolia* and, to our knowledge, in an orchid species (Cameron, 1999; Freudenstein, 2025). We identified 22 *VpGYF* genes organized as 11 homologous pairs across the two haplotype genomes, with comparable gene family size to eight GYF proteins in *Arabidopsis* (Wu et al., 2017; Matsui et al., 2017). The expansion from 8 *Arabidopsis GYF* genes to 22 in vanilla likely reflects the whole-genome duplication in vanilla’s ancestry. Polyploidy often results in redundant gene copies, some of which can diverge and acquire new or specialized functions. In vanilla, the retention of all duplicated *GYF* genes suggests they confer adaptive advantages, perhaps providing robustness to translational control mechanisms or allowing subfunctionalization between paralogs (Hasing et al., 2020).

Phylogenetic analysis resolves the vanilla GYF family into three major clades that mirror the functional lineages previously defined in *Arabidopsis thaliana*, indicating deep evolutionary conservation of GYF-associated regulatory roles across angiosperms (Pontier et al., 2012; Hashimoto et al., 2016a). Clade I comprises the SET–GYF fusion proteins, including *VpGYF1/VpGYF9* and *VpGYF4/VpGYF12*, which are orthologous to *Arabidopsis ATXR3* (*SDG2*) and *ATXR7* (*SDG25*), respectively. In *Arabidopsis*, these proteins regulate defense-associated transcription through H3K4 methylation and chromatin remodeling (Berr et al., 2010; Lee et al., 2016). The conservation of this clade in vanilla suggests that epigenetic control of immune-related gene expression predates the monocot–dicot split; however, the limited transcriptional responsiveness of these genes to CymMV infection indicates that they likely contribute to basal immune regulation rather than acting as inducible susceptibility factors. Clade III encompasses the *EXA1*-like proteins, including *VpGYF3/VpGYF11* and *VpGYF5/VpGYF13*, and represents the most prominent lineage-specific expansion in vanilla relative to *Arabidopsis*, which harbors a single *EXA1* gene. EXA1 has been reported to function as a susceptibility factor for potexvirus infection in *Arabidops*is by facilitating viral RNA translation through interaction with eIF4E-family cap-binding proteins (Hashimoto et al., 2016b; Nishikawa et al., 2023). Within this clade, several genes showed infection-associated transcriptional responses following CymMV inoculation, although the strongest induction across the VpGYF family was observed in other loci such as *VpGYF4/GYF12*, *VpGYF19/GYF21*, and *VpGYF6/GYF14.* This expansion may reflect gene dosage effects or subfunctionalization following orchid whole-genome duplication, potentially enhancing host factor availability exploited during potexvirus infection. Clade II contains the *NERD-*like GYF proteins, which in *Arabidopsis* integrate RNA-based pathways with epigenetic regulation through interactions with small RNA machinery and DNA methylation processes (Pontier et al., 2012). The presence of multiple *NERD*-like orthologs in vanilla underscores conservation of this regulatory axis; however, their relatively stable expression following CymMV challenge suggests that these genes function primarily in housekeeping or developmental regulation rather than as active components of virus-induced susceptibility. Collectively, these clade-specific patterns indicate that CymMV-responsive transcriptional changes occur across multiple *GYF* lineages in vanilla, rather than being confined to a single clade.

### *VpGYF* expression correlates with vanillin accumulation

RNA-seq analysis revealed unexpected tissue- and accession-specific *VpGYF* expression patterns implicating these genes in secondary metabolism. The high-vanillin accession consistently exhibited elevated *VpGYF* expression relative to the low-vanillin accession across tissues and developmental stages. In this study, *VpGYF* expression correlated with vanillin accumulation capacity across tissues and accessions, a pattern suggesting that GYF-mediated post-transcriptional regulation could influence secondary metabolism, including vanillin biosynthesis, although this association is likely indirect and context-dependent. This interpretation is consistent with the established role of GYF proteins in mRNA surveillance and translational control (Ash et al., 2010). White tissue, inner mesocarp, endocarp, placenta, and seeds, where glucovanillin accumulates at >300 mM (Gastelbondo et al., 2025) exhibited preferential *VpGYF* expression, consistent with the spatial concentration of vanillin biosynthetic machinery. GYF proteins function in mRNA surveillance and translational control (Ash et al., 2010), processes critical when cells sustain massive metabolic flux while managing cytotoxic intermediates. Elevated *VpGYF* expression in AC85 likely reflects enhanced post-transcriptional quality control during active secondary metabolism.

Temporal dynamics differed between tissues: green tissue expression increased from month 6 to 8 as outer layers transitioned toward senescence; white tissue expression declined over this interval, indicating transient peaks during active glucovanillin accumulation followed by stabilization. Hierarchical clustering identified *VpGYF8/VpGYF16* and *VpGYF7/VpGYF15* as white-tissue–enriched pairs that resist this decline, candidate regulators of vanillin accumulation capacity and potential molecular breeding targets.

### VpGYF expression dynamics parallel viral accumulation: evidence for susceptibility factor function

The family-wide transcriptional responsiveness of *VpGYF* genes following CymMV inoculation represents a central finding of this study. When recalculated using the comparative Ct (ΔΔCt) method and visualized as log2 fold change relative to time-matched controls, *VpGYF* loci showed heterogeneous response magnitudes across the nine-week time course. *VpGYF4/GYF12*, *VpGYF19/GYF21*, and *VpGYF6/GYF14* displayed the strongest and most sustained infection-associated induction, while other loci exhibited moderate or transient profiles. These patterns suggest functional partitioning within the VpGYF family during CymMV challenge and are consistent with potential roles for GYF-domain proteins in virus–host interactions described in other potexvirus systems; however, functional validation is required to determine which loci directly contribute to viral replication or symptom development in vanilla. In contrast, *Arabidopsis* exhibits constitutive, moderate *EXA1* expression under basal conditions (Hashimoto et al., 2016a). This difference indicates either species-specific transcriptional regulation or distinct dynamics during potexvirus infection in orchids.

Across the *VpGYF* family, CymMV infection elicited heterogeneous transcriptional responses with both response magnitude and timing varying among loci. The strongest and most sustained induction was observed for *VpGYF4/GYF12*, *VpGYF19/GYF21*, and *VpGYF6/GYF14*, which exhibited elevated expression across multiple sampling points during the infection time course. Other loci displayed moderate or transient expression increases, suggesting functional differentiation among *VpGYF* family members during viral challenge. These patterns indicate that CymMV responsiveness is distributed across several *GYF* lineages rather than confined to a single ortholog group, consistent with potential roles for multiple GYF-domain proteins in virus–host interactions. Visible symptoms consistently trailed viral accumulation; a threshold viral load is required before physiological disruption becomes apparent (Pagán and García-Arenal, 2018). Early *VpGYF* induction could facilitate initial replication; later spikes near peak viral load may reflect systemic spread. These patterns extend findings from other potexvirus systems. *Arabidopsis EXA1* is essential for PlAMV infection; *exa1* loss-of-function mutants exhibit near-complete resistance (Hashimoto et al., 2016a). VIGS of *NbEXA1* in *N. benthamiana* compromised the accumulation of multiple potexviruses (Yusa et al., 2019). Our CymMV accumulation data (**Figure 8**), which reached ~30, 000 relative transcript abundance units by Week 9, further support the association between *VpGYF* responsiveness and viral multiplication. These observations suggest that *VpGYF* genes may participate in host pathways associated with viral multiplication, although functional validation will be required to confirm this role in vanilla.

Mechanistically, *EXA1*-like proteins serve as accessory factors for host translational machinery. The recruitment of eIF4E-family translation factors to viral RNA indicates that Arabidopsis *EXA1* engages these cap-binding proteins through its conserved Y-X^4^-L-ϕ interaction motif (Nishikawa et al., 2023). VpGYFs likely have an analogous function, licensing the host translation system for CymMV multiplication. Some *VpGYF* loci exhibited temporally dynamic expression profiles over the infection time course, which may reflect shifting host demands on RNA metabolism and translation during different stages of infection. Similar EXA1-dependent virus susceptibility mechanisms have been described in Arabidopsis and Nicotiana species, and homologous GYF-domain proteins are also present in crops such as rice and tomato, suggesting that conserved host translational regulatory pathways may influence virus–host interactions across diverse plant lineages.

### The susceptibility factor paradox: why does the host upregulate genes that benefit the virus?

The strong induction observed in several *VpGYF* loci during CymMV infection, likely reflects a convergence of host and viral processes rather than a single causal mechanism. On one hand, the strong temporal correlation between *VpGYF* expression and viral accumulation supports viral co-optation, as potexviruses require *EXA1* function for efficient multiplication and enhanced expression of this susceptibility factor would directly facilitate viral RNA translation and replication (Yusa et al., 2019; Hyodo and Okuno, 2014; Sanfaçon, 2015). On the other hand, *EXA1*-like GYF proteins participate in mRNA surveillance and translational homeostasis, and their induction may also represent a host stress response to the increased translational burden imposed by viral RNA, an effect that could inadvertently benefit the virus (Matsui et al., 2017; Sanfaçon, 2015). These interpretations are not mutually exclusive and may operate simultaneously during infection. In addition, GYF proteins can exert context-dependent antiviral functions, as exemplified by *PSIG1*-mediated restriction of pathogen-induced cell death through interaction with *SMG7* (Matsui et al., 2017), and prior work has demonstrated that members of the GYF family can have both pro-viral and antiviral roles depending on cellular context (Hashimoto et al., 2016a). The hierarchical expression pattern observed here, involving multiple *VpGYF* loci including *VpGYF4/GYF12, VpGYF19/GYF21*, and *VpGYF6/GYF14*, likely reflects functional partitioning within the family, wherein some members may contribute to virus–host interactions while others participate in RNA surveillance, silencing pathways, or broader regulatory processes associated with antiviral defense (Baulcombe, 2022; Cao et al., 2014).

### Engineering virus resistance: targets and considerations

Genes showing the strongest infection-associated induction, including *VpGYF4/GYF12, VpGYF19/GYF21*, and *VpGYF6/GYF14*, represent promising candidates for functional analysis in the context of host factor–based virus resistance in vanilla. In *Arabidopsis*, exa1 knockouts display near-complete resistance to multiple potexviruses without major growth penalties (Hashimoto et al., 2016a). By analogy, loss-of-function mutations in infection-responsive *VpGYF* loci could potentially reduce CymMV susceptibility, which would be particularly valuable in vanilla where conventional breeding is constrained by vegetative propagation and limited genetic diversity (Truniger and Aranda, 2009; Gu et al., 2024; Bory et al., 2008). However, functional redundancy within the expanded *GYF* family may complicate this strategy. Multiple *VpGYF* genes show coordinated expression patterns, raising the possibility of compensatory effects; consequently, single knockouts may be insufficient to produce a strong resistance phenotype (Kafri et al., 2016; Wu et al., 2017; Ali et al., 2015; Xing et al., 2014). The distinct temporal expression profiles observed across *VpGYF* loci during infection, including the strongest induction in *VpGYF4/GYF12*, *VpGYF19/GYF21*, and *VpGYF6/GYF14*, provide additional candidates and timing cues for prioritizing targets for functional testing (García-Ordóñez and Pagán, 2024).

The apparent involvement of *VpGYF* genes in both fruit-associated processes and infection-associated responses raises important considerations for crop improvement. Any host factor–based resistance strategy should be evaluated for potential effects on fruit development and vanillin-related metabolism (Gallage et al., 2014).

### Limitations and future directions

Several limitations of the present study should be acknowledged. In particular, regulatory relationships inferred from promoter motif analysis and predicted protein–protein interaction networks represent computational hypotheses that require experimental validation. First, our conclusions are based primarily on expression profiling and orthology-based inference rather than direct functional validation. While transcriptional induction and phylogenetic conservation provide strong evidence for involvement in virus–host interactions, expression data alone cannot establish causality. Previous studies in Arabidopsis and other systems have demonstrated that loss-of-function mutations in susceptibility factors such as *EXA1* are required to definitively confirm their role in viral replication (Hashimoto et al., 2016a; Yusa et al., 2019). Similarly, correlational associations between gene expression and secondary metabolism have been widely used for hypothesis generation but require experimental validation to confirm regulatory function (Usadel et al., 2009; Rao and Dixon, 2019). A second limitation relates to technical constraints in vanilla functional genomics. Stable genetic transformation and targeted mutagenesis in orchids remain challenging and the lack of routine, high-efficiency transformation systems has historically limited functional studies in *Vanilla planifolia* and related species (Truniger and Aranda, 2009; Huang et al., 2012). Although recent advances in genome assembly and tissue culture have expanded molecular resources for vanilla (Hasing et al., 2020; Piet et al., 2022), functional validation of candidate genes remains a bottleneck. To address these limitations, functional validation of *VpGYF* genes through targeted mutagenesis represents a critical next step. In particular, CRISPR/Cas-mediated knockout of infection-responsive *VpGYF* loci, including the strongly induced candidates identified in this study, would directly test their roles in CymMV susceptibility, while multiplex editing strategies may be required to resolve potential functional redundancy within the family (Hashimoto et al., 2016a; Xing et al., 2014; Ali et al., 2015). Complementary approaches such as transient expression assays, protein–protein interaction assays, and validation in heterologous systems may also help clarify gene function where stable transformation remains difficult. In addition, this study suggests that distinct *VpGYF* family members may partition roles between developmental regulation and stress responses. Future work should therefore assess potential trade-offs between virus resistance and secondary metabolism by evaluating vanillin accumulation, fruit development, and plant fitness in edited lines. Such analyses are particularly important in vanilla, where economic value is tightly linked to metabolite production (Gallage et al., 2014; Gastelbondo et al., 2025).

Finally, mechanistic studies examining protein–protein interactions will be essential to confirm whether vanilla *EXA1*-like proteins engage the host translational machinery through the same conserved Y-X^4^-L-ϕ motif described in Arabidopsis (Nishikawa et al., 2023). Co-immunoprecipitation, yeast two-hybrid assays, and *in vivo* localization studies could clarify whether VpGYF proteins interact with eIF4E-family members and RNA silencing components during CymMV infection. Extension of these analyses to additional orchid species would further determine whether the regulatory patterns identified here represent orchid-wide strategies or lineage-specific adaptations.

## Conclusions

We present the first genome-wide characterization of the *GYF* gene family in *Vanilla planifolia* and, to our knowledge, in an orchid species, partitioned into functionally distinct clades corresponding to Arabidopsis proteins involved in translational control, chromatin modification, and RNA silencing. *VpGYF* expression during fruit development was associated with vanillin accumulation capacity. Broad transcriptional responsiveness following CymMV infection, together with expression waves that parallel viral accumulation and symptom progression, suggests that members of the family may participate in host–potexvirus interactions. The strongest infection-associated transcriptional induction was observed for *VpGYF4/GYF12, VpGYF19/GYF21*, and *VpGYF6/GYF14*, nominating these loci as priority candidates for functional validation and potential host factor–based resistance strategies. This work establishes a genomic and functional framework for future studies aimed at improving vanilla production through enhanced disease resistance.

## Data Availability

The datasets presented in this study can be found in online repositories. The RNA-seq data used for fruit transcriptome analysis are available in the NCBI Sequence Read Archive under BioProject accession number PRJNA974693.
