# Supplementary material for: Genome-wide identification and expression profiling of the GYF gene family in Vanilla planifolia: insights into fruit development and Cymbidium mosaic virus response
Source: Front Plant Sci. 2026 Apr 17;17:1795375. doi: 10.3389/fpls.2026.1795375 (PMC13132833; doi:10.3389/fpls.2026.1795375)
Supplement: Supplementary file 1 [file DataSheet1.docx]

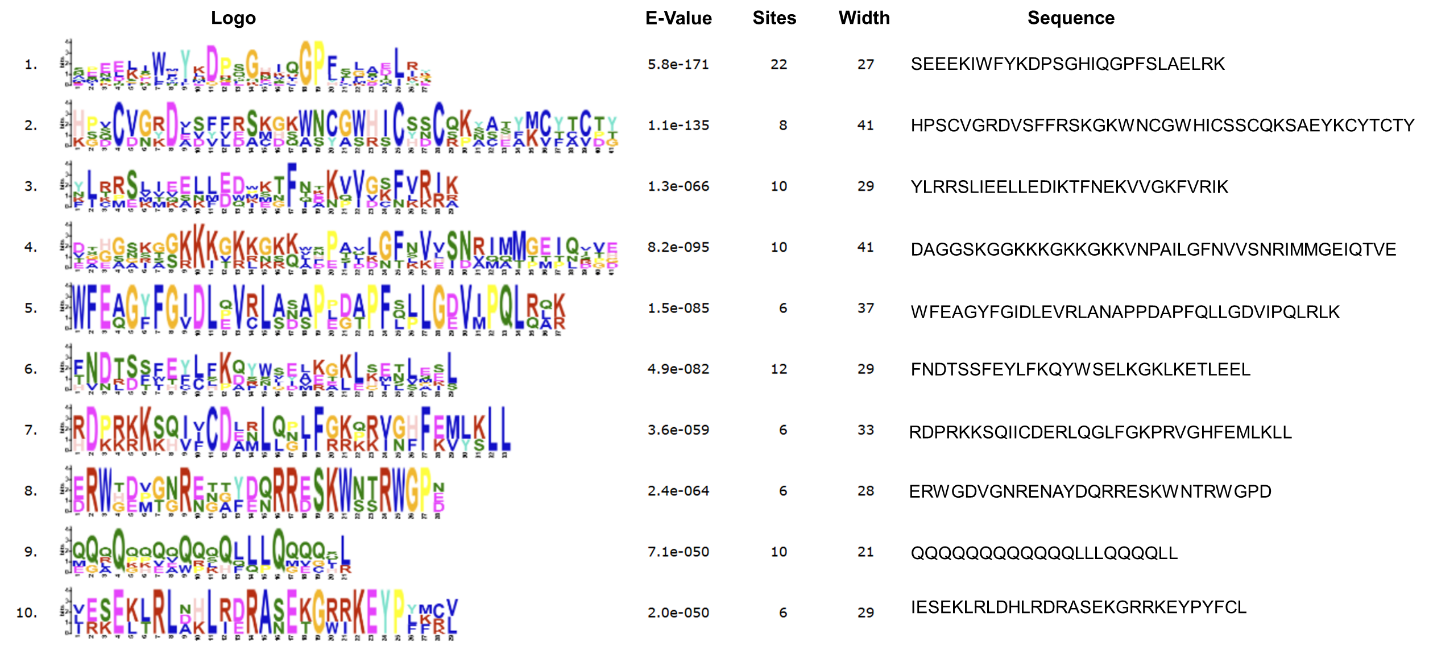


**Supplemental Fig. 1** Conservation analysis of VpGYF protein motifs. Sequence logos of ten conserved motifs in GYF proteins generated using MEME Suite. The height of each letter represents the relative frequency of the corresponding amino acid at that position, with taller letters indicating higher conservation. The columns to the right of each motif provide additional information.


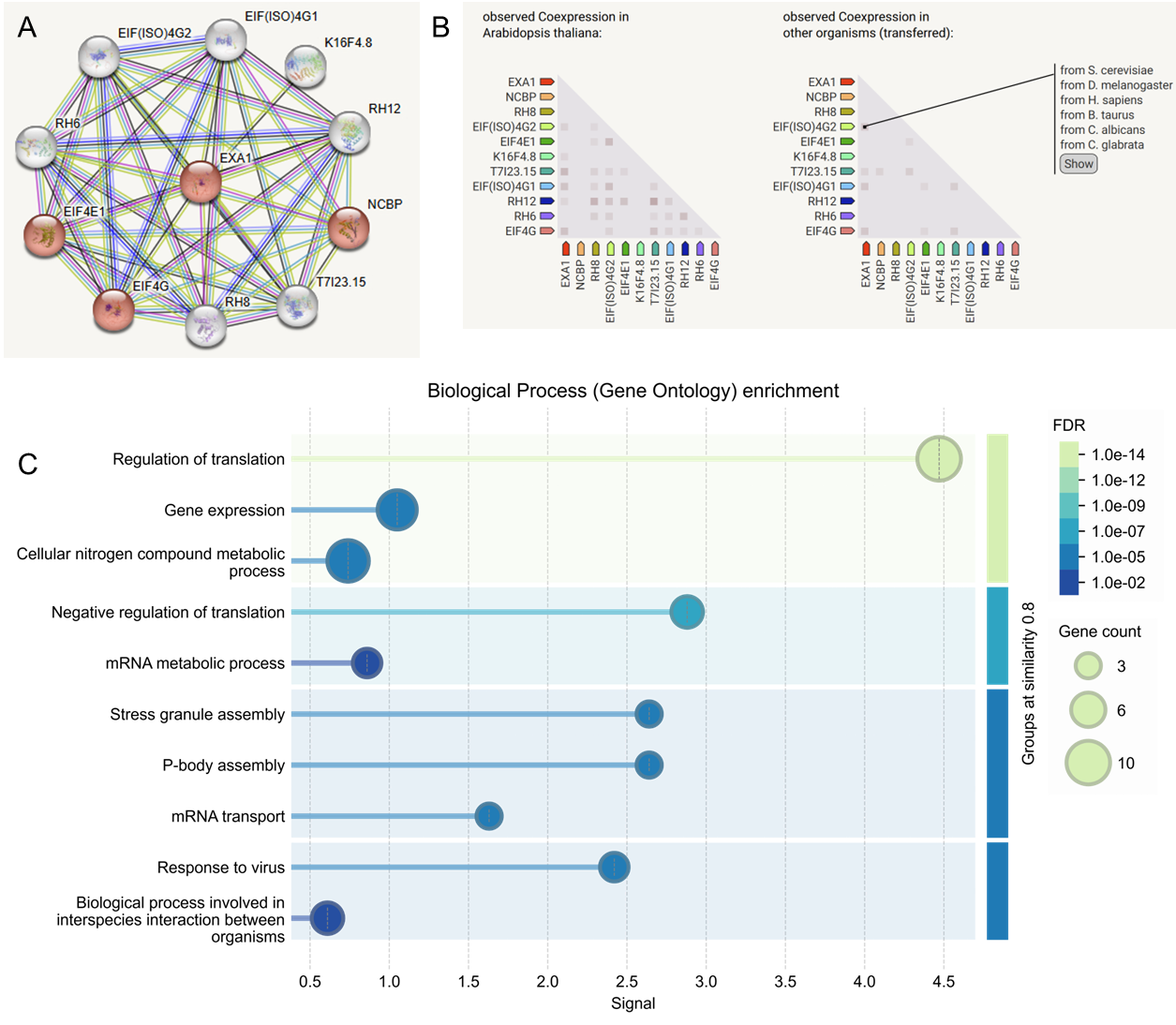


**Supplemental Fig. 2** Protein-protein interaction network and functional enrichment of At*EXA1*-associated proteins. (A) At*EXA1*-centered protein-protein interaction (PPI) network in *Arabidopsis thaliana* predicted by STRING. Nodes represent individual proteins, and edges indicate interactions supported by experimental evidence, curated databases, and computational prediction. Edge thickness reflects interaction confidence, integrating information from text mining, co-expression, and homology; genes labeled in red are involved in virus responses. (B) Co-expression patterns of *AtEXA1* and its network partners in *A. thaliana* and other organisms, as inferred from STRING co-expression matrices. (C) GO enrichment analysis of *AtEXA1* network genes, showing fold enrichment for significantly over-represented biological processes, molecular functions, and cellular components; circle size denotes gene count, and color indicates FDR-adjusted significance.

**Supplemental Table 1 Protein properties and functions of *VpGYF* family**

| Gene Name | Molecular Weight | Theoretical PI | Instability Index | Aliphatic Index | Grand Average of Hydropathicity | Pfam | Pos | Localization |
| --- | --- | --- | --- | --- | --- | --- | --- | --- |
| VpGYF1 | 267042.58 | 6.5 | 52.98 | 68.39 | -0.78 | PF02213/  PF14237 | 942..986  543..588,942..987 | Nuclear |
| VpGYF2 | 55370.43 | 4.8 | 41.89 | 81.32 | -0.543 | PF02213/  PF14237 | 17..61  17..66 | Cytoplasmic |
| VpGYF3 | 191879.17 | 6.28 | 54.83 | 66.69 | -0.735 | PF02213 | 571..605 | Nuclear |
| VpGYF4 | 140209.01 | 6.46 | 47.21 | 82.85 | -0.366 | PF02213/  PF14237 | 58..91, 204..245  60..91 | Nuclear |
| VpGYF5 | 208453.63 | 6.23 | 53.38 | 70.01 | -0.728 | PF02213 | 599..635 | Nuclear |
| VpGYF6 | 199584 | 5.22 | 51.92 | 68.28 | -0.605 | PF02213/  PF14237 | 1173..1211  1173..1196 | Nuclear |
| VpGYF7 | 161319.59 | 6.23 | 51.64 | 64.06 | -0.71 | PF02213/  PF14237 | 860..898  860..896 | Nuclear |
| VpGYF8 | 185059.16 | 5.44 | 52.43 | 67.29 | -0.813 | PF02213 | 559..606 | Nuclear |
| VpGYF9 | 267013.6 | 6.47 | 53.03 | 68.64 | -0.776 | PF02213/  PF14237 | 942..986  543..588,942..987 | Nuclear |
| VpGYF10 | 55370.43 | 4.8 | 41.89 | 81.32 | -0.543 | PF02213/  PF14237 | 17..61  17..66 | Cytoplasmic |
| VpGYF11 | 192865.32 | 6.28 | 53.98 | 67.07 | -0.73 | PF02213 | 580..614 | Nuclear |
| VpGYF12 | 138537.96 | 6.4 | 47.84 | 82.35 | -0.384 | PF02213/  PF14237 | 42..75, 188..229  44..75 | Nuclear |
| VpGYF13 | 208238.35 | 6.22 | 53.64 | 70.34 | -0.728 | PF02213 | 598..634 | Nuclear |
| VpGYF14 | 199487.05 | 5.22 | 52.57 | 69.5 | -0.587 | PF02213/  PF14237 | 1173..1211  1173..1196 | Nuclear |
| VpGYF15 | 160884.89 | 6.25 | 52.03 | 63.4 | -0.733 | PF02213/  PF14237 | 856..894  856..892 | Nuclear |
| VpGYF16 | 185587.77 | 5.46 | 52.87 | 67.29 | -0.818 | PF02213 | 559..606 | Nuclear |
| VpGYF17 | 91296.47 | 5.78 | 37.83 | 74.49 | -0.675 | PF02213 | 742..780 | Nuclear |
| VpGYF18 | 91296.47 | 5.78 | 37.83 | 74.49 | -0.675 | PF02213 | 742..780 | Nuclear |
| VpGYF19 | 57761.14 | 9.3 | 34.83 | 71.67 | -0.639 | PF14237 | 244..294 | Mitochondrial |
| VpGYF20 | 285946 | 5.9 | 46.86 | 97.61 | -0.082 | PF14237 | 1202..1252 | Nuclear/  Cytoplasmic/  PlasmaMembrane |
| VpGYF21 | 57761.14 | 9.3 | 34.83 | 71.67 | -0.639 | PF14237 | 244..294 | Mitochondrial |
| VpGYF22 | 285946 | 5.9 | 46.86 | 97.61 | -0.082 | PF14237 | 1202..1252 | Nuclear/  Cytoplasmic/  PlasmaMembrane |

**Supplemental Table 2 Ks, Ka, and Ka/Ks, type of mutation, and duplication of *VpGYF* gene pairs**

| Paralog 1 | Paralog 2 | Ks | Ka | Ka/Ks | Duplication | Type of Mutation/Evolution | p-value |
| --- | --- | --- | --- | --- | --- | --- | --- |
| VpGYF1 | VpGYF2 | 1.828 | 1.286 | 0.704 | SD | Negative or Purifying |  |
| VpGYF1 | VpGYF3 | 1.797 | 1.599 | 0.890 | SD | Negative or Purifying |  |
| VpGYF1 | VpGYF4 | 1.448 | 1.839 | 1.270 | SD | Positive or Diversifying |  |
| VpGYF1 | VpGYF5 | 2.019 | 2.132 | 1.056 | SD | Positive or Diversifying |  |
| VpGYF1 | VpGYF7 | 2.056 | 1.555 | 0.756 | SD | Negative or Purifying |  |
| VpGYF1 | VpGYF8 | 1.802 | 2.080 | 1.154 | SD | Positive or Diversifying |  |
| VpGYF1 | VpGYF10 | 1.828 | 1.286 | 0.704 | SD | Negative or Purifying |  |
| VpGYF1 | VpGYF11 | 1.770 | 1.605 | 0.907 | SD | Negative or Purifying |  |
| VpGYF1 | VpGYF12 | 1.448 | 1.839 | 1.270 | SD | Positive or Diversifying |  |
| VpGYF1 | VpGYF13 | 1.959 | 2.126 | 1.085 | SD | Positive or Diversifying |  |
| VpGYF1 | VpGYF15 | 1.953 | 1.547 | 0.792 | SD | Negative or Purifying |  |
| VpGYF1 | VpGYF16 | 1.908 | 2.021 | 1.059 | SD | Positive or Diversifying |  |
| VpGYF1 | VpGYF17 | 2.594 | 1.514 | 0.584 | SD | Negative or Purifying |  |
| VpGYF2 | VpGYF3 | 2.881 | 1.453 | 0.504 | SD | Negative or Purifying |  |
| VpGYF2 | VpGYF4 | 1.589 | 1.370 | 0.862 | SD | Negative or Purifying |  |
| VpGYF2 | VpGYF6 | 1.954 | 1.558 | 0.797 | SD | Negative or Purifying |  |
| VpGYF2 | VpGYF7 | 2.286 | 1.306 | 0.571 | SD | Negative or Purifying |  |
| VpGYF2 | VpGYF9 | 1.828 | 1.286 | 0.704 | SD | Negative or Purifying |  |
| VpGYF2 | VpGYF11 | 2.811 | 1.425 | 0.507 | SD | Negative or Purifying |  |
| VpGYF2 | VpGYF12 | 1.589 | 1.370 | 0.862 | SD | Negative or Purifying |  |
| VpGYF2 | VpGYF14 | 1.942 | 1.560 | 0.803 | SD | Negative or Purifying |  |
| VpGYF2 | VpGYF15 | 2.238 | 1.310 | 0.585 | SD | Negative or Purifying |  |
| VpGYF2 | VpGYF17 | 1.726 | 1.564 | 0.906 | SD | Negative or Purifying |  |
| VpGYF3 | VpGYF4 | 1.851 | 2.089 | 1.128 | SD | Positive or Diversifying |  |
| VpGYF3 | VpGYF5 | 1.972 | 0.766 | 0.389 | SD | Negative or Purifying |  |
| VpGYF3 | VpGYF6 | 2.292 | 1.694 | 0.739 | SD | Negative or Purifying |  |
| VpGYF3 | VpGYF7 | 1.700 | 1.595 | 0.938 | SD | Negative or Purifying |  |
| VpGYF3 | VpGYF9 | 1.797 | 1.599 | 0.890 | SD | Negative or Purifying |  |
| VpGYF3 | VpGYF10 | 2.881 | 1.453 | 0.504 | SD | Negative or Purifying |  |
| VpGYF3 | VpGYF11 | 0.028 | 0.006 | 0.222 | SD | Negative or Purifying |  |
| VpGYF3 | VpGYF12 | 1.851 | 2.089 | 1.128 | SD | Positive or Diversifying |  |
| VpGYF3 | VpGYF13 | 1.904 | 0.770 | 0.405 | SD | Negative or Purifying |  |
| VpGYF3 | VpGYF14 | 2.376 | 1.714 | 0.721 | SD | Negative or Purifying |  |
| VpGYF3 | VpGYF15 | 1.849 | 1.588 | 0.859 | SD | Negative or Purifying |  |
| VpGYF3 | VpGYF17 | 3.209 | 1.602 | 0.499 | SD | Negative or Purifying |  |
| VpGYF4 | VpGYF5 | 2.243 | 2.204 | 0.983 | SD | Negative or Purifying |  |
| VpGYF4 | VpGYF6 | 1.638 | 1.908 | 1.165 | SD | Positive or Diversifying |  |
| VpGYF4 | VpGYF7 | 2.499 | 1.748 | 0.700 | SD | Negative or Purifying |  |
| VpGYF4 | VpGYF8 | 2.167 | 1.731 | 0.799 | SD | Negative or Purifying |  |
| VpGYF4 | VpGYF9 | 1.448 | 1.839 | 1.270 | SD | Positive or Diversifying |  |
| VpGYF4 | VpGYF10 | 1.589 | 1.370 | 0.862 | SD | Negative or Purifying |  |
| VpGYF4 | VpGYF11 | 1.960 | 2.075 | 1.059 | SD | Negative or Purifying |  |
| VpGYF4 | VpGYF13 | 2.394 | 2.226 | 0.930 | SD | Negative or Purifying |  |
| VpGYF4 | VpGYF14 | 1.613 | 1.899 | 1.177 | SD | Positive or Diversifying |  |
| VpGYF4 | VpGYF15 | 2.595 | 1.739 | 0.670 | SD | Negative or Purifying |  |
| VpGYF4 | VpGYF16 | 2.075 | 1.730 | 0.834 | SD | Negative or Purifying |  |
| VpGYF4 | VpGYF17 | 2.114 | 1.596 | 0.755 | SD | Negative or Purifying |  |
| VpGYF5 | VpGYF7 | 2.608 | 1.595 | 0.612 | SD | Negative or Purifying |  |
| VpGYF5 | VpGYF9 | 2.019 | 2.132 | 1.056 | SD | Positive or Diversifying |  |
| VpGYF5 | VpGYF11 | 1.945 | 0.763 | 0.393 | SD | Negative or Purifying |  |
| VpGYF5 | VpGYF12 | 2.243 | 2.204 | 0.983 | SD | Negative or Purifying |  |
| VpGYF5 | VpGYF13 | 0.011 | 0.003 | 0.280 | SD | Negative or Purifying |  |
| VpGYF5 | VpGYF15 | 2.563 | 1.586 | 0.619 | SD | Negative or Purifying |  |
| VpGYF5 | VpGYF16 | 3.140 | 1.138 | 0.363 | SD | Negative or Purifying |  |
| VpGYF5 | VpGYF17 | 2.008 | 1.457 | 0.726 | SD | Negative or Purifying |  |
| VpGYF6 | VpGYF7 | 1.437 | 0.448 | 0.312 | SD | Negative or Purifying |  |
| VpGYF6 | VpGYF10 | 1.954 | 1.558 | 0.797 | SD | Negative or Purifying |  |
| VpGYF6 | VpGYF11 | 2.498 | 1.686 | 0.675 | SD | Negative or Purifying |  |
| VpGYF6 | VpGYF12 | 1.638 | 1.908 | 1.165 | SD | Positive or Diversifying |  |
| VpGYF6 | VpGYF15 | 1.322 | 0.447 | 0.338 | SD | Negative or Purifying |  |
| VpGYF6 | VpGYF17 | 1.590 | 0.819 | 0.515 | SD | Negative or Purifying |  |
| VpGYF7 | VpGYF9 | 2.056 | 1.555 | 0.756 | SD | Negative or Purifying |  |
| VpGYF7 | VpGYF10 | 2.286 | 1.306 | 0.571 | SD | Negative or Purifying |  |
| VpGYF7 | VpGYF11 | 1.664 | 1.578 | 0.948 | SD | Negative or Purifying |  |
| VpGYF7 | VpGYF12 | 2.499 | 1.748 | 0.700 | SD | Negative or Purifying |  |
| VpGYF7 | VpGYF13 | 3.171 | 1.592 | 0.502 | SD | Negative or Purifying |  |
| VpGYF7 | VpGYF14 | 1.462 | 0.449 | 0.307 | SD | Negative or Purifying |  |
| VpGYF7 | VpGYF15 | 0.028 | 0.005 | 0.163 | SD | Negative or Purifying |  |
| VpGYF7 | VpGYF17 | 1.865 | 0.818 | 0.439 | SD | Negative or Purifying |  |
| VpGYF8 | VpGYF9 | 1.802 | 2.080 | 1.154 | SD | Positive or Diversifying |  |
| VpGYF8 | VpGYF12 | 2.167 | 1.731 | 0.799 | SD | Negative or Purifying |  |
| VpGYF8 | VpGYF15 | 3.052 | 1.443 | 0.473 | SD | Negative or Purifying |  |
| VpGYF8 | VpGYF16 | 0.068 | 0.006 | 0.089 | SD | Negative or Purifying |  |
| VpGYF8 | VpGYF17 | 2.881 | 1.649 | 0.572 | SD | Negative or Purifying |  |
| VpGYF9 | VpGYF10 | 1.828 | 1.286 | 0.704 | SD | Negative or Purifying |  |
| VpGYF9 | VpGYF11 | 1.770 | 1.605 | 0.907 | SD | Negative or Purifying |  |
| VpGYF9 | VpGYF12 | 1.448 | 1.839 | 1.270 | SD | Positive or Diversifying |  |
| VpGYF9 | VpGYF13 | 1.959 | 2.126 | 1.085 | SD | Negative or Purifying |  |
| VpGYF9 | VpGYF15 | 1.953 | 1.547 | 0.792 | SD | Negative or Purifying |  |
| VpGYF9 | VpGYF16 | 1.908 | 2.021 | 1.059 | SD | Positive or Diversifying |  |
| VpGYF9 | VpGYF17 | 2.594 | 1.514 | 0.584 | SD | Negative or Purifying |  |
| VpGYF10 | VpGYF11 | 2.811 | 1.425 | 0.507 | SD | Negative or Purifying |  |
| VpGYF10 | VpGYF12 | 1.589 | 1.370 | 0.862 | SD | Negative or Purifying |  |
| VpGYF10 | VpGYF14 | 1.942 | 1.560 | 0.803 | SD | Negative or Purifying |  |
| VpGYF10 | VpGYF15 | 2.238 | 1.310 | 0.585 | SD | Negative or Purifying |  |
| VpGYF10 | VpGYF17 | 1.726 | 1.564 | 0.906 | SD | Negative or Purifying |  |
| VpGYF11 | VpGYF12 | 1.960 | 2.075 | 1.059 | SD | Positive or Diversifying |  |
| VpGYF11 | VpGYF13 | 1.880 | 0.768 | 0.408 | SD | Negative or Purifying |  |
| VpGYF11 | VpGYF14 | 2.611 | 1.706 | 0.653 | SD | Negative or Purifying |  |
| VpGYF11 | VpGYF15 | 1.806 | 1.571 | 0.870 | SD | Negative or Purifying |  |
| VpGYF11 | VpGYF17 | 2.472 | 1.644 | 0.665 | SD | Negative or Purifying |  |
| VpGYF12 | VpGYF13 | 2.394 | 2.226 | 0.930 | SD | Negative or Purifying |  |
| VpGYF12 | VpGYF14 | 1.613 | 1.899 | 1.177 | SD | Positive or Diversifying |  |
| VpGYF12 | VpGYF15 | 2.595 | 1.739 | 0.670 | SD | Negative or Purifying |  |
| VpGYF12 | VpGYF16 | 2.075 | 1.730 | 0.834 | SD | Negative or Purifying |  |
| VpGYF12 | VpGYF17 | 2.114 | 1.596 | 0.755 | SD | Negative or Purifying |  |
| VpGYF13 | VpGYF15 | 3.082 | 1.583 | 0.514 | SD | Negative or Purifying |  |
| VpGYF13 | VpGYF16 | 3.237 | 1.137 | 0.351 | SD | Negative or Purifying |  |
| VpGYF13 | VpGYF17 | 2.209 | 1.454 | 0.659 | SD | Negative or Purifying |  |
| VpGYF14 | VpGYF15 | 1.344 | 0.448 | 0.333 | SD | Negative or Purifying |  |
| VpGYF14 | VpGYF17 | 1.605 | 0.822 | 0.512 | SD | Negative or Purifying |  |
| VpGYF15 | VpGYF17 | 2.028 | 0.821 | 0.405 | TM | Negative or Purifying |  |
| VpGYF16 | VpGYF17 | 3.018 | 1.616 | 0.535 | TM | Negative or Purifying |  |

| Gene ID | Primer Pair | Forward Primer | Tm | Reverse Primer | Tm | Length |  |
| --- | --- | --- | --- | --- | --- | --- | --- |
| VPCR0040H1_01G018200 | VpGYF1/VpGYF9 | TGATGGCAGTTGTGTTTCGG | 59 | CTGTCTGGACTAGGTAGCTCC | 59 | 75 |  |
| VPCR0040H2_01G024290 | VpGYF1/VpGYF9 |  | | | | | |
| VPCR0040H1_05G011030 | VpGYF2/VpGYF10 | CAATTCCTGAGGTGCTTGCC | 59 | CCTCTTGCTGCCATTTCACA | 59 | 102 |  |
| VPCR0040H2_05G012040 | VpGYF2/VpGYF10 |  |  |  |  |  |  |
| VPCR0040H2_09G020020 | VpGYF3/VpGYF11 | GCCGTTTCTGATATCGCGTT | 59 | GGAACTGTTGCTTTGACACG | 58 | 83 |  |
| VPCR0040H1_09G013090 | VpGYF3/VpGYF11 |  |  |  |  |  |  |
| VPCR0040H2_10G012540 | VpGYF4/VpGYF12 | CAGATCTGTAGACTCCTTTCCGA | 59 | AACAACAGATGCCACACCAG | 59 | 91 |  |
| VPCR0040H1_10G015910 | VpGYF4/VpGYF12 |  |  |  |  |  |  |
| VPCR0040H1_13G006070 | VpGYF5/VpGYF13 | CTGAACGTGTGGTCTCAAGG | 58 | CGAGCAGGGGAGGTAAGAAG | 58 | 77 |  |
| VPCR0040H2_13G006130 | VpGYF5/VpGYF13 |  |  |  |  |  |  |
| VPCR0040H2_15G002570 | VpGYF6/VpGYF14 | AAGTCCAAACGTAGAGGCCA | 59 | CAGTGGCATCTTTAGCAGGC | 59 | 92 |  |
| VPCR0040H1_15G002400 | VpGYF6/VpGYF14 |  |  |  |  |  |  |
| VPCR0040H2_16G000480 | VpGYF7/VpGYF15 | CTCACCACCGCAATTACCT | 57 | GGCTCGTTTGACTGATAGGT | 57 | 83 |  |
| VPCR0040H1_16G000240 | VpGYF7/VpGYF15 |  |  |  |  |  |  |
| VPCR0040H2_16G006880 | VpGYF8/VpGYF16 | GCAAAGAGCGGCAAGAAGAA | 58 | TTCAAGGTCCACAGCAATCG | 58 | 80 |  |
| VPCR0040H1_16G011600 | VpGYF8/VpGYF16 |  |  |  |  |  |  |
| VPCR0040H2_16G011320 | VpGYF17/ VpGYF18 | TCCCAAAGAGAACAGTGAAGGA | 59 | CACAGACCTCAATAGTGCAATCA | 59 | 77 |  |
| VPCR0040H1_16G016220 | VpGYF17/ VpGYF18 |  |  |  |  |  |  |
| VPCR0040H2_15G007020 | VpGYF19/VpGYF21 | AGGTTTCCTCATCTTCCTCCTC | 59 | TACCAGACTTCTCCTCCTCTAC | 58 | 75 |  |
| VPCR0040H1_15G007110 | VpGYF19/VpGYF21 |  |  |  |  |  |  |
| VPCR0040H1_07G011340 | VpGYF20/GYF22 | ATGGATTTTGTCAGTCGGCA | 58 | ATGCAACGGTAACAGGACG | 58 | 109 |  |
| VPCR0040H2_07G017390 | VpGYF20/GYF22 |  |  |  |  |  |  |

**Supplemental Table 3. Primers used for qRT-PCR analysis of *VpGYF* family**
